# Protein lipidation in health and disease: molecular basis, physiological function and pathological implication

**DOI:** 10.1038/s41392-024-01759-7

**Published:** 2024-03-15

**Authors:** Yuan Yuan, Peiyuan Li, Jianghui Li, Qiu Zhao, Ying Chang, Xingxing He

**Affiliations:** 1 Department of Gastroenterology, Tongji Hospital, Tongji Medical College, Huazhong University of Science and Technology, Wuhan, China; 2 Department of Gastroenterology, Zhongnan Hospital of Wuhan University, Wuhan, China; 3 Hubei Clinical Center and Key Laboratory of Intestinal and Colorectal Diseases, Wuhan, China

**Keywords:** Molecular biology, Diseases

## Abstract

Posttranslational modifications increase the complexity and functional diversity of proteins in response to complex external stimuli and internal changes. Among these, protein lipidations which refer to lipid attachment to proteins are prominent, which primarily encompassing five types including S-palmitoylation, N-myristoylation, S-prenylation, glycosylphosphatidylinositol (GPI) anchor and cholesterylation. Lipid attachment to proteins plays an essential role in the regulation of protein trafficking, localisation, stability, conformation, interactions and signal transduction by enhancing hydrophobicity. Accumulating evidence from genetic, structural, and biomedical studies has consistently shown that protein lipidation is pivotal in the regulation of broad physiological functions and is inextricably linked to a variety of diseases. Decades of dedicated research have driven the development of a wide range of drugs targeting protein lipidation, and several agents have been developed and tested in preclinical and clinical studies, some of which, such as asciminib and lonafarnib are FDA-approved for therapeutic use, indicating that targeting protein lipidations represents a promising therapeutic strategy. Here, we comprehensively review the known regulatory enzymes and catalytic mechanisms of various protein lipidation types, outline the impact of protein lipidations on physiology and disease, and highlight potential therapeutic targets and clinical research progress, aiming to provide a comprehensive reference for future protein lipidation research.

## Introduction

**Fig. 1:**
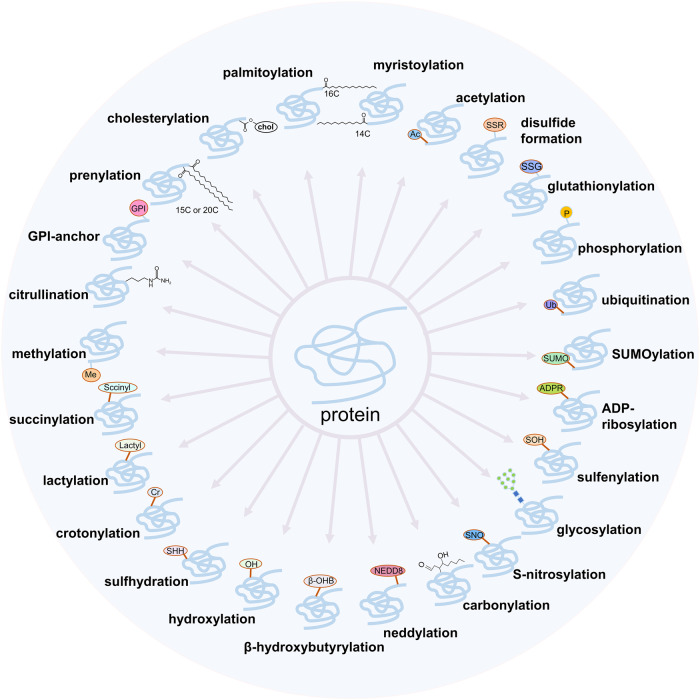
The main commonly studied posttranslational modifications (PTMs) that are associated with various diseases. PTMs play a critical role in a broad spectrum of biological processes. Here, we conclude that 25 common types of PTMs and dysregulation of those PTMs are strongly associated with the pathogenesis of a variety of diseases

Over billions of years, organisms have acquired mechanisms, such as alternative splicing, cotranslational modifications and posttranslational modifications (PTMs), that allow genomes containing 20,000–25,000 genes to be converted into proteomes of more than one million proteins, thereby enhancing the complexity and functional diversity of proteins in response to complex external stimuli and internal changes.^1^ PTMs are covalent or enzymatic modifications that occur at the amino acid side chain of proteins after their biosynthesis. There are various types of PTMs, such as the addition of functional/chemical groups, the addition of polypeptide chains, the addition of complex molecules, and the modification of amino acids.^2^ To date, over 620 types of PTMs have been identified to regulate protein activity, and at least 20 PTMs have been extensively studied (Fig. 1) and found to be strongly associated with the pathogenesis of a variety of diseases.^3^

**Fig. 2:**
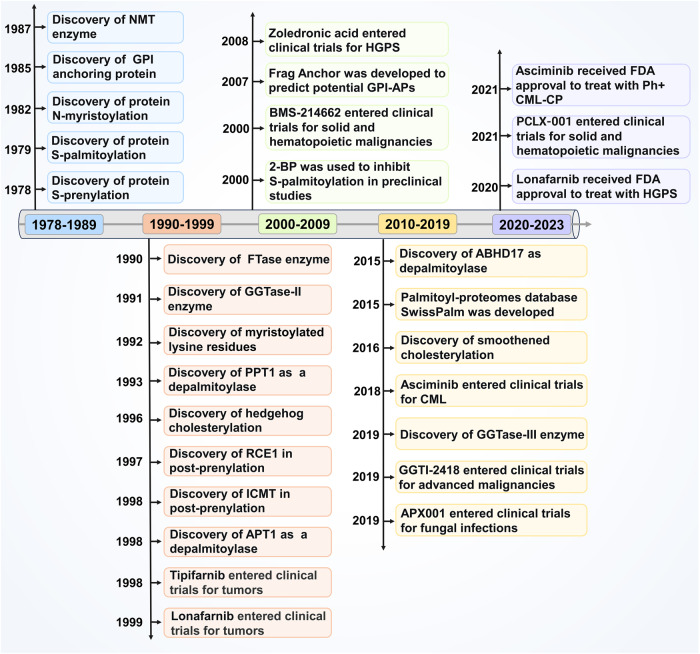
The timeline of protein lipidation discoveries. The figure illustrates the timeline of protein lipidation research from their origin to the most advanced scientific discoveries, including regulatory enzymes, important protein substrates, clinical trials and the development of inhibitors

Protein lipidation, one of the most common PTMs, can attach up to seven different types of lipids, including fatty acids (FA), lipoic acids, isoprenoids, sterols, phospholipids, glycosylphosphatidylinositol anchors, and lipid-derived electrophiles, to proteins.^4^ Based on the location of the modified proteins, protein lipidation can be divided into two categories. The first category comprises lipid modifications that occur in the cytoplasm or on the cytoplasmic side of membranes, including S-palmitoylation, N-myristoylation and S-prenylation. The other category is composed of lipid modifications that occur in the lumen of secretory organelles, such as glycosylphosphatidylinositol (GPI) anchor and cholesterylation. The 16-carbon fatty acid palmitate covalently linked to cysteine (Cys) residues of proteins via the labile thioester bond is defined as protein S-palmitoylation.^5^ Protein S-palmitoylation was first reported in 1979 and is catalysed by DHHC palmitoyl S-acyltransferases (DHHC-PATs), while depalmitoylation is mediated by depalmitoylases.^6–9^ Given the instability of the thioester bond, S-palmitoylation is characterised as the typical reversible type of lipidation.^10^ Techniques for detecting protein S-palmitoylation include radioactive isotope-labelled palmitic acid, click chemistry-based metabolic labelling with bioorthogonal palmitic acid probes, acyl-biotin exchange and others.^11^ In addition, there are other less frequently occurring palmitoylation types. O-palmitoylation is characterised by the attachment of palmitate to serine residues. N-palmitoylation is characterised by the attachment of palmitate to the N-terminus.^12^ The attachment of 14-carbon myristic acid to N-terminal glycine residues via an amide bond in a manner of co- or posttranslational modification is referred to as N-myristoylation.^13,14^ Protein N-myristoylation was first reported in 1982,^15^ and is catalysed by N-myristoyltransferase (NMT). Approaches for detecting protein N-myristoylation include radioactive-labelled fatty acids and bioorthogonal probes such as azido or alkynyl myristate analogues.^14^ Canonical N-myristoylation occurring on glycine residues is irreversible, but when myristic acid is attached to lysine residues, fatty acyl groups from Nε-modified Lys residues can be removed by sirtuins and histone deacetylases (HDACs),^16,17^ suggesting that lysine myristoylation is another reversible lipidation following S-palmitoylation. S-prenylation refers to the attachment of a 15-carbon farnesyl or a 20-carbon geranylgeranyl isoprenoid lipid onto Cys residues of proteins via a thioether bond.^18^ Protein S-prenylation was first reported in fungi in 1978 and later in mammals,^19,20^ and is mainly catalysed by protein farnesyltransferase (FTase) or protein geranylgeranyltransferase I (GGTase-I). Protein S-prenylation can be detected by bioorthogonal probes such as alkynyl isoprenoid probes and azido-isoprenoid probes.^14^ Most prenylated proteins belong to the small GTPase family, including the Ras subfamily, the Rab subfamily, the Rho subfamily and others.^21^ Generally, the C-terminal CAAX motif of substrate proteins is the structural basis for enzyme recognition, and after the Cys residue is prenylated, the -AAX residues are removed by RCE1, followed by a methyl group transfer to the isoprenoid-modified Cys residue by ICMT.^22^ The GPI anchor refers to the amino group of EtNP at the end of GPI attached to the carboxyl group at the C-terminus of the protein via an amide bond. During translocation from the endoplasmic reticulum (ER) to the plasma membrane (PM), nascent GPI-anchoring proteins (GPI-APs) undergo several remodelling steps on the immature GPI structure before maturation and finally anchor to the outer leaflet of the lipid bilayer.^23^ Detection of GPI-APs can be achieved by chemical probes such as a bifunctional analogue probe of the conserved glucosaminylphosphatidylinositol motif.^24^ To date, it is estimated that more than 250 GPI-APs are present in eukaryotes with various functions, including enzymes, receptors, antigens, and adhesion molecules, therefore mediating a myriad of biological functions.^25^ Cholesterylation refers to the covalent attachment of cholesterol to proteins in an autoprocessing manner,^26^ which can be labelled and detected by azido- and alkyne-modified cholesterol analogues.^14^ Currently known cholesterylated proteins include hedgehog (Hh) and smoothened (SMO), which are key molecules in the Hh signalling pathway and regulate a variety of important physiological activities such as embryonic development.^27^ The timeline of discovery of the five lipidation types is exhibited in Fig. 2.

Lipid attachment to proteins plays a crucial role in regulating protein trafficking, localisation, stability, conformation, interactions, and signal transduction by enhancing the hydrophobicity of proteins, which serve as fundamental regulators of protein physiological functions. An increasing number of studies have revealed that aberrant regulation of lipidation is implicated in the pathogenesis of various diseases, such as cancer, neurological disorders, cardiovascular diseases, inflammatory diseases, metabolic disorders, infectious diseases and others, suggesting that elucidating the role of lipidation regulation in disease progression is promising for the development of potential therapeutic targets. In this review, we discuss the known regulatory enzymes and catalytic mechanisms of various lipidation types, outline the role of protein lipidation in physiology and disease, and highlight potential therapeutic targets and clinical research progress. We systematically review the relevant advances in protein lipidation with the aim of providing a comprehensive protein lipidation reference.

## S-palmitoylation

### Regulatory enzymes and catalytic mechanism

S-palmitoylation is catalysed by a specific class of enzymes called DHHC-PATs, which share a common structural feature of a highly conserved Asp–His–His–Cys tetrapeptide motif within a cysteine-rich region, which is where the catalytic reaction occurs.^8,28^ To date, 23 DHHC-PATs have been identified in *Homo sapiens* and several different DHHC proteins have been found in other species.^29^ As a type of polytopic integral membrane protein, DHHC-PATs possess 4-7 transmembrane domains (TMDs) and mainly localise to the Golgi and ER, while a minority localise to the PM, mitochondria and perineuclears regions.^30,31^ The localisation of DHHC-PATs partly depends on structure and motif. For example, lysine-based sorting signals on the sequences of DHHC4 and DHHC6 enable them to form KXX and KKXX motifs, thus guiding them to the ER membrane.^32^ DHHC1, 2, 4, 9, 12, 14, 20, and 22 can traffic between the ER and Golgi.^33^ Among DHHC-PATs, DHHC20 is most widely distributed in the cytosol, including PM, ER, Golgi and perineuclears regions.^31,34,35^

**Fig. 3:**
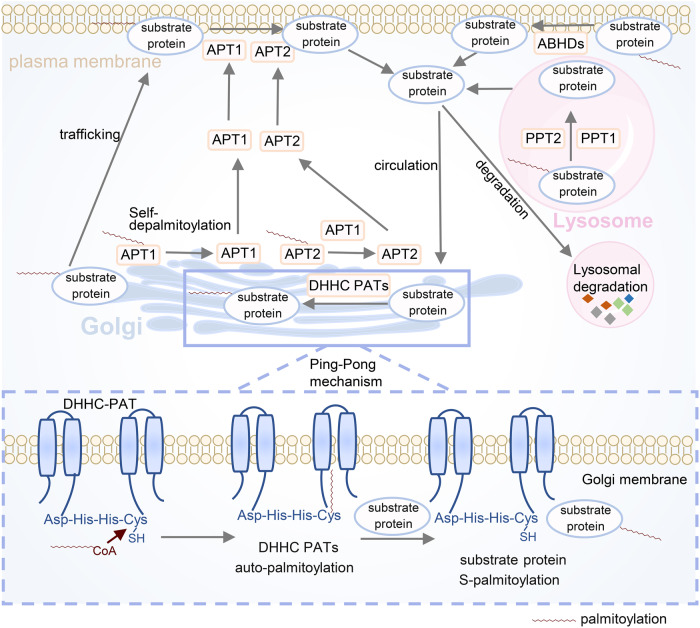
Catalytic mechanisms of DHHC palmitoyl S-acyltransferases (DHHC-PATs) and depalmitoylases. Some DHHC-PATs go through two steps (ping-pong kinetic mechanisms) to catalyse substrate protein S-palmitoylation. First, DHHC-PAT undergoes autopalmitoylation, linking palmitoyl-CoA to Cys156 in the DHHC domain to form an acyl-enzyme transfer intermediate. Subsequently, the palmitoyl chain is transferred to the putative cysteine residue on the substrate protein to complete S-palmitoylation. For depalmitoylases, three categories of depalmitoylases are subject to being palmitoylated to arrive at proper positions to function. A portion of palmitoylated APT1 and APT2 localise to the Golgi, and APT1 is capable of depalmitoylating itself and APT2, which allows them to be released into the cytosol to ensure the steady-state distribution of APTs in the Golgi and the cytosol. A large proportion of palmitoylated proteins are depalmitoylated by APT1 and APT2 on the plasma membrane or in the cytosol. Some palmitoylated proteins on the plasma membrane could also be depalmitoylated by ABHDs. PPT1 and PPT2 are mainly responsible for depalmitoylation of substrate proteins that localise in lysosomes. There are two fates for substrate proteins after depalmitoylation. One of them returns to catalytic positions, such as the Golgi, to undergo repalmitoylation, and the other enters the lysosome to be degraded

Although 23 DHHC-PATs are known to be responsible for catalysing protein S-palmitoylation, the exact process remains obscure. Previous studies have found that some DHHC-PATs go through two steps to catalyse substrate S-palmitoylation (ping-pong kinetic mechanisms)^36^ (Fig. 3). First, DHHC-PAT undergoes autopalmitoylation, in which the thiolate nucleophile of Cys156 attacks the carbonyl carbon in the palmitoyl-CoA thioester to form an acyl-enzyme transfer intermediate that links palmitoyl-CoA to Cys156 in the DHHC domain. Subsequently, the palmitoyl chain is transferred to the putative cysteine residue on the substrate protein to complete S-palmitoylation.^35,37^ However, autopalmitoylation of DHHC13, 17, 19 and 22 was not detectable by tritiated palmitate or alkyne fatty acid analogues,^38–40^ and more investigations are needed to explore whether the autopalmitoylation of these enzymes are not possible with other detection techniques or whether autopalmitoylation does not occur in all DHHC-PATs. Although the DHHC domain provides the key catalytic core and fatty acid binding cavity, understanding the mechanism of S-palmitoylation is clouded by exploring the role of the conserved DHHC domain alone. For instance, in addition to the DHHC domain, other conserved motifs such as Asp–Pro–Gly (DPG), Thr–Thr-x–Glu (TTxE), and PaCCT (palmitoyltransferase conserved C- terminus) have been reported to be adjacent to transmembrane domains.^8^ The second threonine of the TTxE motif could directly interact with the aspartic acid of the DHHC motif to form a hydrogen bond, and Asn266 in the PaCCT motif exploits its H-bond capabilities to assist the side chain in forming an amide, indicating that those conserved residues are potentially involved in substrate protein recognition and the catalytic process through some crucial contacts with the DHHC domain.^35^ In addition, some DHHC-PATs require interactions with cofactors to form complexes, such as the DHHC9/GCP16 complex^41^ and DHHC6/Selk complex,^42^ or need to be palmitoylated by other DHHC family members to provoke S-palmitoylation cascades before regulating substrate S-palmitoylation.^43,44^ Moreover, Robyn Stix et al. conducted an all-atom molecular dynamics simulation experiment and discovered that during the catalytic process, hDHHC20 induced local deformation of membranes, especially in the cytosolic site where cysteine is catalytic, allowing cysteine to be optimally positioned for autopalmitoylation with fatty acyl-CoA.^45^ However, whether this enzyme-induced membrane deformation and the adjustment of the catalytic site position are common phenomena in the palmitoylation process remain to be further explored in other DHHC-PATs. In addition, although the sequence for the S-palmitoylation site has not yet reached a consensus, some deep learning-based palmitoylation site prediction websites, such as GPS-Palm, can provide important clues for researchers to explore various palmitoylation sites,^46^ which immensely aids the study of protein S-palmitoylation.

The functional redundancy of DHHC-PATs as well as substrate redundancy complicates the resolution of individual DHHC characteristics.^47,48^ It remains unclear whether the recognition sites of the enzyme-substrate interaction, as well as the catalytic sites where S-palmitoylation occurs, are the same when the protein substrate can be palmitoylated by multiple DHHC-PATs. The degree of binding specificity between DHHC-PAT and its substrates has attracted the attention of researchers. Some unique protein‒protein interaction motifs, such as PDZ domains in substrate proteins and ankyrin repeats and the SH3 domain in DHHC-PATs, may provide docking capabilities to participate in substrate selectivity.^49^ For instance, the S-palmitoylation of the multi-PDZ domain-containing protein GRIP1b by DHHC5/8 requires a PDZ ligand-dependent recognition mechanism, as well as the S-palmitoylation of PSD93 by DHHC14.^49,50^ In addition, ankyrin repeats in the N-terminus of DHHC13 and DHHC17 and the type II PDZ-binding motif of DHHC6 may participate in substrate recruitment and regulation.^51^ The fusion of ankyrin repeats to the N-terminus of DHHC3 endows DHHC3 with the ability to regulate the S-palmitoylation of huntingtin proteins and their trafficking, which would otherwise not occur.^51^ Although enzyme functional and substrate redundancy has hindered the identification of individual DHHC-PATs to some extent, redundancy exists so that substrate proteins can be regulated by corresponding DHHC-PATs in different compartments or single DHHC-PATs can catalyse a fair amount of substrates to meet physiological needs.^52^ In turn, the localisation of DHHC-PATs in different organelles is also involved in substrate selectivity.

To date, there is still an absence of small-molecule probes or drugs that specifically inhibit DHHC-PATs. Among various lipid-based covalent inhibitors that paninhibit DHHC-PATs, 2-bromopalmitate (2-BP) is the most widely used and irreversibly inhibits S-palmitoylation by occupying the lipid binding cavity of the DHHC protein.^35,53^ Nevertheless, there are many concerns about using 2-BP to study the physiological or pathological role of S-palmitoylation, as in addition to inhibiting protein S-palmitoylation, 2-BP also regulates lipid synthesis, transport, and metabolism and even inhibits the depalmitoylases acyl protein thioesterase 1 (APT1) and APT2.^48,54^ To overcome the main shortcomings of 2-BP, Tong Lan et al. developed a novel broad-spectrum DHHC inhibitor cyanomethyl-N-myracrylamide (CMA), and they found that significant inhibition of the S-palmitoylation level in protein substrates was achieved by lower concentrations of CMA than 2-BP, and that CMA did not disturb the activities of APT1 and APT2.^54^ However, there is a paucity of direct experimental data on CMA that support these findings. Agents that selectively inhibit DHHC-PAT must be developed to overcome the current shortcomings in the study of S-palmitoylation.

Protein depalmitoylation is mainly achieved by cleavage of the thioester bond on palmitoylated proteins. Enzymes responsible for regulating depalmitoylation can be divided into three categories, namely APTs, palmitoyl‐protein thioesterases (PPTs) and ABHD17 family thioesterases.^9^ They all belong to the ‘metabolic’ serine hydrolase superfamily, with an active site serine for substrate hydrolysis and the ability to cleave ester, amide, or thioester bonds on small molecules, peptides and proteins.^55,56^ APT1 was initially recognised for its capability to hydrolyse a variety of lysophospholipids. It was not until 1998 that Duncan et al. discovered that it could remove radiolabelled palmitate from the α‐subunit of G‐proteins and other proteins, and it had a stronger depalmitoylation activity.^57^ It is generally believed that APT1 mainly localises to the cytosol, but it has also been reported that APT1 can localise to the PM, Golgi, ER, mitochondria and even the nuclear membrane to perform its catalytic function.^58–60^ APT2 is another depalmitoylase primarily localised in the cytosol and shares nearly 60% identity with APT1.^55^ Previous reports have shown that both APT1 and APT2 are palmitoylated at the Cys residue at position 2. APT1 is capable of depalmitoylating itself and APT2, rendering both APT1 and APT2 dynamically palmitoylated.^61^ The subcellular localisation and depalmitoylation activity of APTs are closely linked to the regulation of APT itself by dynamic palmitoylation.^61^ Vartak et al. proposed that due to dynamic palmitoylation regulation, a substantial fraction of palmitoylated APT1 and APT2 is recruited to the Golgi apparatus, whereas they undergo depalmitoylation in trans, resulting in their release into the cytosol to ensure the steady-state distribution of APTs in the Golgi and cytosol, and the depalmitoylation of palmitoylated proteins in various parts of the membrane is regulated by nonpalmitoylated APTs, which is crucial for substrate proteins to be repalmitoylated to execute their functions.^61^ Although these enzymes share approximately 60% identity, studies suggest that there are preferences for specific palmitoylated substrates between APT1 and APT2. For example, APT1, but not APT2, reverses agonist-dependent S-palmitoylation of β2-adrenergic receptors.^62^ The depalmitoylation of GAP-43 and DHHC6 depends on APT2 rather than APT1.^43,63^ The structure of apo-APT1 was first reported as early as 2000, but it was not until 2016 that the high-resolution structure of the complex formed by the binding of APT1/2 to their respective inhibitors ML348 and ML349 was resolved, contributing to resolving the functional differences between APT1 and APT2.^64^ These enzymes include unique structures such as the β5-α2 loop.^64^ The β5-α2 loop and residues around the catalytic triad contribute to the formation of hydrophobic channels that regulate the entry and engagement of hydrophobic substrates of different lengths by altering the openness, which is irrelevant to the selectivity of palmitoylated substrates.^64^ More in-depth studies are needed to explore the factors involved in the selectivity of palmitoylated substrates.

In addition to cytosolic APTs, another class of enzymes PPTs (PPT1 and PPT2), localise in lysosomes and are involved in protein depalmitoylation. PPT1 was the first enzyme found to have the function to depalmitoylate protein.^65^ Normally, PPT1 targets lysosomes via the mannose 6-phosphate receptor-mediated pathway and facilitates the degradation of substrate proteins by depalmitoylating them in the lysosomes.^66–68^ Previously, it was thought that PPT1 depalmitoylation activity plays a major part in protein degradation in lysosomes.^69^ However, Erica et al. found that in the PPT1 knockout mouse model, most substrate proteins exhibited an increase in palmitoylation, which was accompanied by unchanged or decreased protein levels, indicating that the depalmitoylation activity of PPT1 was not entirely for protein degradation.^70^ PPT2 is another lysosomal thioesterase homologous to PPT1 and shares 26% identity with PPT1.^71^ PPT2 does not have thioesterase activity for some substrates such as S-palmitoyl H-Ras. The crystal structures of PPT1 and PPT2 reveal that although both have similar architectural features, conformational differences near the active site partially explain substrate selectivity, as the entrance space of the lipid binding site consisting of β3-αA and β8-αF in PPT1 is larger than that in PPT2 and thus more inclusive of the substrates.^71^ Deficiency of PPT1 or PPT2 results in serious neurodegeneration, while overexpression of PPT1 or PPT2 may accelerate tumour growth.^72–75^

ABHDs including ABHD10, ABHD12 and ABHD17A/B/C have been identified as another category of cytosolic depalmitoylases.^76^ ABHD10 was found to modulate the depalmitoylation of the key antioxidant protein PRDX5, and ABHD12 could reduce PSD95 S-palmitoylation.^9,77^ ABHD17 proteins mainly regulate the depalmitoylation of proteins on the PM.^78^ With the subsequent identification of other substrate proteins, the possibility that ABHD17 regulates the depalmitoylation of substrates at other locations cannot be ruled out. ABHD17 also undergoes S-palmitoylation, which facilitates its targeting of postsynaptic membranes and enhancement of enzymatic activity.^9^ ABHD17 has a dual function. On the one hand, it promotes the depalmitoylation of the substrate protein PSD95 for dynamic palmitate turnover on proteins. On the other hand, it reduces the autopalmitoylation of DHHC proteins, increasing the complexity of the palmitoylation regulatory network.^79^ ABHD family proteins and APTs exhibit partial substrate redundancy. However, there are limited reports on the depalmitoylase activity, substrate-binding structure features, enzyme kinetics, substrate preferences and other physiological roles of ABHD family proteins. More efforts are needed to deepen the understanding of depalmitoylases for the development of corresponding small-molecule drugs for treatment.

Both broad-spectrum and selective inhibitors are used to inhibit the catalytic activity of despalmitoylases. Palmostatin B, a classical broad-spectrum inhibitor, has been shown to block the activities of APT1/2, PPT1, ABHD17A/B/C and other lipid-processing serine hydrolases with varying potency.^9,80^ Palmostatin M, another inhibitor structurally related to Palmostatin B, has similar biochemical IC50 values but is more active in cells.^81^ ML348 and ML349 are piperazine amide competitive inhibitors of APT1 and APT2, respectively. Structurally, ML348 is located above the catalytic triad and blocks it by contacting hydrophobic residues.^64^ ML349 blocks the function of APT2 by indirectly contacting the catalytic triad with the formation of a hydrogen bond network through the sulfur group and the water molecule at the catalytic active site.^64^ Selective inhibitors targeting the depalmitoylation activity of PPTs are not available. Recently, two autophagy inhibitors, GNS561 and hydroxychloroquine (HCQ), were shown to inhibit PPT1 and thus target lysosomes for cancer therapy.^75,82^ Intriguingly, inhibition of PPT1 with HCQ increased the palmitoylation level of AEG-1, which is regulated by PPT1/2, demonstrating that HCQ is a potential inhibitor of the depalmitoylation activity of PPT1.^83^ Regarding selective inhibitors that inhibit the ABHD17 enzyme, although ABD957 may act as a selective covalent inhibitor of the ABHD17 enzyme compared with traditional broad-spectrum inhibitors according to MS-ABPP data and in situ time-course ABHD17A/B/C inhibition experiments, it partially rescued the palmitoylation level of N-Ras.^78^ Future efforts are needed to elucidate the localisation and interaction patterns of ABHD17 enzymes and perform detailed biochemical and phenotypic analyses of each isoform, which could also benefit the development of relevant selective inhibitors.

### Physiological function

#### Protein trafficking and membrane localisation

S-palmitoylation is capable of regulating protein trafficking and localisation, the hydrophobic palmitate covalently attached to the protein acts as a lipid anchor to increase protein lipophilicity and facilitate its interaction with the membrane.^84^ The dynamic palmitoylation-depalmitoylation cycle facilitates the trafficking of substrate proteins between the Golgi, PM, and endosomal recycling system by modulating their hydrophobicity.^5^ For example, the fatty acid transporter CD36 (also known as the scavenger receptor) is a widely expressed membrane glycoprotein with various metabolic functions, such as the regulation of fatty acid uptake.^85^ S-palmitoylation increases the incorporation of CD36 into the PM by enhancing its hydrophobicity, whereas inhibition of S-palmitoylation leads to CD36 accumulation on the ER.^86^ DHHC4 and DHHC5 were found to mediate CD36 S-palmitoylation, with DHHC4 responsible for CD36 S-palmitoylation at the Golgi, followed by vesicle-mediating trafficking of CD36 to the PM, while DHHC5 is responsible for keeping CD36 at the PM.^87^ Another study discovered that during the process of fatty acid uptake, CD36 was under the control of dynamic S-palmitoylation regulation.^88^ This was mainly reflected in the downstream kinase LYN being activated to phosphorylate DHHC5 when fatty acids interact with CD36.^88^ Phosphorylated DHHC5 loses its catalytic capacity, and CD36 is further depalmitoylated by APT1 to initiate endocytic uptake of FA.^88^

Proper localisation of proteins, mediated by S-palmitoylation in specific subcellular compartments, directly influences cell polarity. The apical‒basal polarity of epithelial cells regulates their proliferation, apoptosis, and migration.^89^ Loss of cell polarity leads to tissue disorganisation, abnormal proliferation and migration, and transformation of epithelial cells towards cancer.^89^ SCRIB, as a cell junction localisation protein, is a major regulator of epithelial cell polarity.^89^ A double mutation at Cys4 and Cys10 (C4/10S) completely blocked SCRIB S-palmitoylation and caused a diffuse distribution of SCRIB in the cytosol rather than its proper localisation in cell‒cell junctions, which resulted in luminal structure disruption and partial loss of cell polarity.^90^ In addition to epithelial cells, neurons are also highly polarised cells. In neurons, S-palmitoylation regulates synaptic activity and the targeted trafficking of cytosolic proteins to axons and dendrites.^91^ In regard to synaptic activity regulated by S-palmitoylation, as an example, research on the S-palmitoylation of AMPARs, an ionotropic glutamate receptor crucial to synaptic transmission and synaptic plasticity, gradually elucidated the complex regulation of S-palmitoylation.^84^ AMPAR has four subunits, GluA1-4, each with two palmitoylation sites in TMD2 and the C-terminus.^92^ S-palmitoylation at TMD2 increases receptor internalisation and accumulation in the Golgi, and depalmitoylation at this site promotes AMPAR trafficking from the Golgi to the cell surface.^92^ The S-palmitoylation of the C-terminal site regulates the interaction of AMPAR with 4.1 N, and depalmitoylation increases the affinity with AMPAR-associated proteins such as 4.1 N to maintain AMPAR on the cell surface.^92^ Interestingly, the majority of AMPAR-associated proteins, including PSD95, PICK1, GRIP/ABP, AKAP79/150 and others, are subject to S-palmitoylation, but S-palmitoylation of these proteins often produces opposite effects to those of the self-palmitoylation of AMPAR. Both AMPAR and PSD95 are substrates of DHHC3, indicating that S-palmitoylation regulates AMPAR trafficking and localisation in a dynamic and complex network. Any disturbances to this network can cause synaptic AMPAR disorders and lead to the occurrence of related diseases.^84^

While many proteins rely on S-palmitoylation for PM localisation, some proteins exhibit the opposite behaviour upon palmitoylation. FLT3 is a receptor tyrosine kinase that is mainly involved in haematopoietic development, and FLT3 internal tandem repeat (FLT3-ITD) is a common mutation in acute myeloid leukaemia (AML).^93^ FLT3-ITD is mainly localised to the ER rather than the PM, but increased PM localisation of FLT3-ITD was found after mutation of the palmitoylation site Cys563 and disruption of its S-palmitoylation, which was subsequently followed by the activation of Akt and ERK as well as sustained STAT5 activation, finally promoting the development of AML.^93^

While S-palmitoylation is known to facilitate the intracellular transport of substrate proteins by increasing their affinity to membranes, the underlying mechanism remains under investigation. Research indicates that after palmitoylation, substrate proteins aggregate in the curved region of the Golgi margin. Other domains of these proteins then interact with sorting adapters. From these interactions, transport vesicles form and mediate the movement of the substrate proteins to various intracellular compartments, and once they arrive at destinations such as newly formed axons, they will be separated from the vesicles and locally retained through depalmitoylation.^5,94,95^ For some proteins, additional lipidation is necessary for protein trafficking and PM localisation. For instance, H-Ras, N-Ras and K-Ras4a undergo S-palmitoylation and S-prenylation.^96^ Among them, H-Ras needs two palmitoyl groups for recycling endosome targeting and subsequent PM localisation. Such additional lipidation further enhances its hydrophobicity and sufficiently ensures the binding of the protein to the membrane.^97^

#### Protein stability and degradation

S-palmitoylation is also implicated in the regulation of protein stability and degradation, and the absence or blockade of S-palmitoylation usually leads to increased protein transport to lysosomes for degradation. The stability of PD-L1, a notable example, was found to be enhanced by S-palmitoylation.^98^ The development of checkpoint blockade therapies targeting PD-1 or PD-L1 is a major breakthrough in overcoming immune tolerance.^99^ Antibodies against PD-L1 block the transduction of inhibitory signals by binding to PD-L1 on the cell surface. However, these antibodies cannot inhibit the intracellular portion or its redistribution to the PM. Han et.al found that S-palmitoylation of PD-L1 is closely related to its stability.^98^ DHHC3 palmitoylated the Cys272 site in the transmembrane domain of PD-L1. Inhibition of PD-L1 depalmitoylation with ML348 or Palmostatin B resulted in an increase in PD-L1 intracellular expression levels, whereas inhibition of S-palmitoylation with 2-BP generated the opposite effect. Blocking the S-palmitoylation of PD-L1 did not affect its interaction with CMTM6, a molecule that facilitates the trafficking of PD-L1 to recycling endosomes, but rather promoted its lysosomal degradation by inducing PD-L1 ubiquitination through the ESCRT-MVB pathway.^98^ Therefore, targeting the S-palmitoylation of PD-L1 may represent a promising immunotherapy. Inhibition of PD-L1 S-palmitoylation can reduce its overall intracellular storage by promoting its degradation.^98^ Combined with antibodies against PD-L1 on the cell membrane, a strong and long-lasting antitumour synergistic effect may be achieved.

In addition to PD-L1, there are a number of proteins that are protected from lysosomal degradation by S-palmitoylation, including Oct4,^100^ NOD2,^101^ CLDN3,^102^ and Fas.^103^ Conversely, S-palmitoylation can also facilitate protein degradation under certain conditions. For instance, in hepatocellular carcinoma (HCC) cells, when the S-palmitoylation of AEG-1 was reduced by a point mutation in the palmitoylation site C75 or knockout of the corresponding palmitoyltransferase DHHC6, the protein degradation rate was significantly reduced. Subsequent mechanistic studies revealed that the AEG-1 variant was weakly bound to the E3 ubiquitin ligase FBXW7, resulting in decreased ubiquitination and increased stability of AEG-1.^83^ The negative regulation of protein stability by S-palmitoylation also occurs in NLRP3. As a component of the inflammasome, NLRP3 is degraded in lysosomes through chaperone-mediated autophagy after being palmitoylated.^104^ In addition, palmitoylated IFNGR1 is also sorted to lysosomes for degradation through binding to AP3D1, one of the subunits of AP3, which is important in cargo sorting and trafficking.^105^ S-palmitoylation exerts a dual effect on the stability of substrate proteins, either promoting or inhibiting their degradation. This duality might stem from how S-Palmitoylation influences protein–protein interactions. S-palmitoylation tends to promote degradation when it enhances the binding of substrate proteins to molecules associated with degradation processes. In contrast, S-palmitoylation exhibits antidegradation effects when it hinders the binding of substrates to degradation-related proteins.

#### Protein conformation and interaction

Beyond regulating protein trafficking, localisation, and stability, S-palmitoylation can also modify the accessibility of vital active sites, altering protein conformation and disrupting interactions with other proteins. A typical example of how S-palmitoylation meditates protein conformation is caspase-6 (CASP6). CASP6, a member of the cysteine protease family that is mainly involved in the regulation of cell apoptosis and the immune response.^106^ By constructing the crystal structure model of CASP6, it was found that CASP6 had four loops that formed the activation site with the central β-sheet.^107^ CASP6 is palmitoylated by DHHC17 at Cys264 on loop 4 and Cys277 near the start of the β-sheet.^108^ After palmitoylation at Cys264, the flexibility of loop 4 was increased, and the interaction between loop 4 and loop 2 became stronger, which led to the blockage of the activation site and increased the distance between the catalytic dyads, which prevented the activation of CASP6 via nucleophilic attack by C163 on D193.^108^ Moreover, during the palmitoylation at Cys277, Cys277 occupies the active site by linking palmitic acid, thereby blocking the activation and dimerisation of CASP6.^108^ In addition, PSD95 is another protein whose conformation and interactions are influenced by S-palmitoylation. S-palmitoylation induces a conformational change from extended to compact PSD95, which enables its direct binding to NMDAR and AMPAR subunits.^109^ DHHC-PATs and depalmitoylases are more readily aggregated into AMPAR nanodomains, making PSD95 S-palmitoylation and conformation more dynamic when interacting with AMPAR.^109^ Conversely, the state of PSD95 is more stable in the NMDAR nanodomains due to the strong interaction between PSD95 and SAP97, demonstrating that the precise regulation of PSD95 by S-palmitoylation in separate postsynaptic density domains plays an important role in synaptic plasticity.^109^ STING is a key sensor for cytosolic DNA-triggered immune responses, and it has been confirmed in multiple studies that S-palmitoylation is needed for STING activation in the Golgi and type I IFN responses.^110,111^ Recently, a study revealed that STING governs tumour growth through its interaction with VDAC2 on mitochondria, independent of classical innate immunity.^112^ Both STING and VDAV2 can be palmitoylated, while only cysteine mutations in STING but not VDAV2 significantly affect their interaction.^112^

#### Signal transduction

**Fig. 4:**
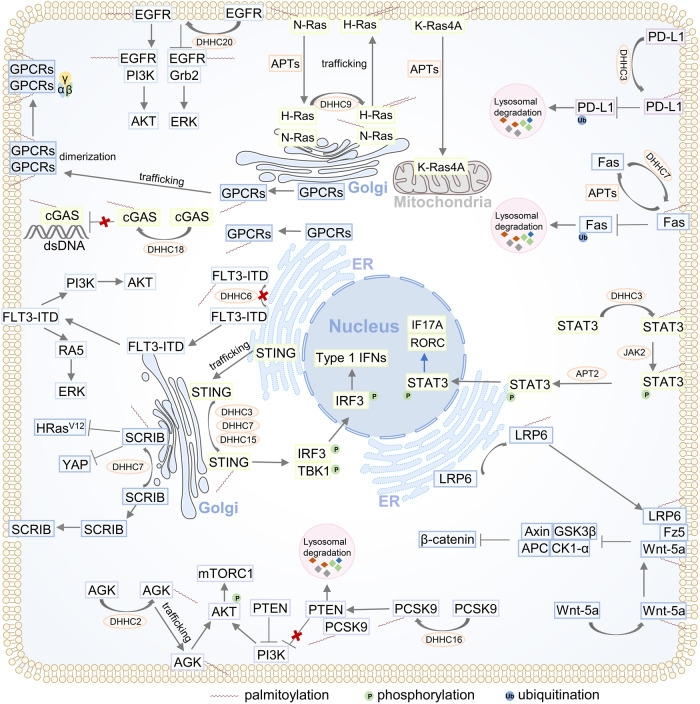
S-palmitoylation is involved in the regulation of multiple cellular signal transduction pathways. We summarised several crucial and well-established signalling pathways that are under the control of S-palmitoylation. The majority of proteins are subjected to S-palmitoylation at the ER or Golgi by the respective DHHC-PATs. Some protein substrates could traffic from the ER to the Golgi or Golgi to the plasma membrane after being palmitoylated to activate the corresponding signalling cascades (e.g., EGFR pathway, N-Ras/H-Ras pathway, GPCR pathway, AGK-Akt-mTORC1 pathway, STING pathway and LRP6 pathway). In some cases, protein substrates anchor at the original organelle after being palmitoylated. Once S-palmitoylation is blocked, these protein substrates detach from the original organelle to abnormally activate the corresponding signalling pathway (e.g., SCRIB pathway and FLT3-ITD pathway). In addition to regulating protein trafficking, some proteins are protected from degradation in lysosomes after being palmitoylated to further activate the corresponding signalling pathway (e.g., Fas pathway and PD-L1 pathway). In addition, S-palmitoylation can regulate the activation of signalling pathways by regulating protein‒protein interactions (e.g., the PCSK9-PI3K-Akt pathway). Furthermore, some protein substrates are under dynamic palmitoylation-depalmitoylation regulation, abnormally activating signalling pathways by increasing palmitate turnover (e.g., the STAT3 pathway). ER endoplasmic reticulum

S-palmitoylation plays a role in regulating various cellular signal transduction pathways, primarily by influencing the aforementioned protein functions. At present, the related signalling pathways involved in tumours have received much attention, including the Ras signalling, EGFR signalling, Wnt signalling, Fas/FasL signalling, DNA damage repair, G protein-coupled receptor (GPCR) signalling and multiple death receptor signalling pathways.^113,114^ In addition, increasing evidence has found that S-palmitoylation also plays an important role in innate and adaptive immune signalling, such as the TLR signalling pathway, NLR signalling pathway, cGAS-STING signalling pathway, sensing DNA and RNA pathway, TNF α-TNF receptor 1 signalling pathway, T-cell coreceptor pathway, PD-1/PD-L1 signalling pathway, B-cell signalling and fragment crystalisable receptor (FcRs) signalling pathway.^115^ The role of S-palmitoylation in these signalling pathways has been previously discussed.^113–115^ Here, we discussed the functions of S-palmitoylation in some important signalling pathways to enrich our knowledge and understanding (Fig. 4). For instance, the cGAS-STING pathway is critical for triggering innate immune responses. For STING, palmitoylation is needed for its activation. However, palmitoylation of cGAS meditated by DHHC18 reduces the interaction between cGAS and double-stranded DNA, thereby negatively regulating cGAS-mediated innate immune responses, suggesting the dual effects of palmitoylation on signalling.^116^ Zhang et al. revealed that S-palmitoylation promotes Th17 differentiation during STAT3 signalling. STAT3 is involved in a palmitoylation-depalmitoylation cycle in colitis. DHHC7 palmitoylates STAT3 in the cytosol, which allows STAT3 to traffic and translocate to the PM for subsequent phosphorylation catalysed by JAK2, and then phosphorylated STAT3 is depalmitoylated by APT2, which leads to STAT3 detachment from the PM and transport to the nucleus to activate the downstream genes RORC and IL17A and initiate the differentiation of TH17 cells.^117^ This study demonstrated that S-palmitoylation regulated the trafficking and localisation of STAT3 as a prerequisite for STAT3 phosphorylation.

The overactivation of the PI3K/Akt signalling pathway abnormally regulates cell-cycle progression, cell proliferation and metabolism, which is an important factor in the progression of a variety of tumours and other diseases.^118^ Recently, accumulating evidence demonstrated the role of S-palmitoylation in regulating the PI3K/Akt signalling pathway. For example, both AGK and GRK6 activate the PI3K/Akt signalling pathway by increasing Akt translocation to the PM through S-palmitoylation.^119,120^ In another study, PCSK9 was found to be related to cell proliferation and drug resistance in hepatocellular carcinoma, as the S-palmitoylation of PCSK9 increased its affinity with PTEN and induced it to target lysosomes for degradation, thereby removing the inhibitory effect of PTEN on Akt signalling and leading to abnormal activation of Akt signalling.^121^ These studies indicate that S-palmitoylation might influence signalling pathways indirectly, coordinating with other posttranslational modifications, such as protein phosphorylation. These findings offer new perspectives on the signalling role of S-palmitoylation.

### Pathological implication

#### Cancer

Currently, multiple studies have highlighted the strong association between S-palmitoylation and cancer. This association can be seen in the retrieved results from SwissPalm, a protein S-palmitoylation database. By retrieving 299 previously identified tumour driver genes in SwissPalm,^122,123^ 132 of them were detected at least once in multiple palmitoyl-proteomes from *Homo sapiens*, and 40 of them were detected at least once in palmitoyl-proteomes from other species. Moreover, a number of studies have also reported that S-palmitoylation also acts as a tumour suppressor in the process of tumour progression, and the protumour/antitumour effects of S-palmitoylation are highly variable in different cancers. However, the palmitoylation-regulating enzymes of these cancer-related proteins and how these protein substrates act in tumour development after being abnormally palmitoylated remain to be explored. Here, we discuss the role of S-palmitoylation in different cancer types to provide a glimpse into the relationship between S-palmitoylation and cancer.

Tumour-associated proteins exhibit enhanced S-palmitoylation in various cancer types, which is achieved through three primary mechanisms, including dysregulation of the PAT and APT enzymes, increased palmitic acid levels, or enhanced binding capacity between enzymes and substrate proteins. For example, it was found that DHHC2 was abnormally upregulated in tyrosine kinase inhibitor-resistant clear cell renal cell carcinoma tissues and cells, and the upregulation of DHHC2 increased the S-palmitoylation of AGK, which promoted the translocation of AGK to the PM for further activation of the PI3K/Akt/mTOR pathway, finally leading to TKI resistance.^119^ In addition, in colorectal cancer, the accumulation of palmitic acid caused by ACOX1 depletion contributes to the enhanced S-palmitoylation of β-catenin. S-palmitoylation protects β-catenin against β-Trcp mediated proteasomal degradation and activates DUSP14 by upregulating c-Myc, thereby boosting colorectal cancer progression.^124^ Moreover, an enhanced ability of enzymes to bind proteins was found in epithelial ovarian cancer. Epithelial ovarian cancer relies on the tricarboxylic acid cycle and oxidative phosphorylation to meet its anabolic growth requirements.^125^ S-palmitoylation increases the activity of the key tricarboxylic acid cycle enzyme MDH2 to support mitochondrial respiration and tumour cell proliferation, and the increased S-palmitoylation was attributed to the enhanced binding of DHHC18 to MDH2.^125^

Numerous tumour-associated proteins depend on S-palmitoylation for trafficking and proper localisation to the PM, enabling them to function effectively, either for signalling to activate various oncogenic signalling pathways, as mentioned above, or to assist tumour cells in taking up nutrients to meet their high metabolic demands. For instance, GLUT1 is essential for glucose uptake, and GLUT1 is palmitoylated by DHHC9 at Cys207 before it is accurately localised to the PM for glucose transport. Furthermore, disturbance of GLUT1 S-palmitoylation abrogated tumour glycolysis, thereby hindering the tumorigenesis of glioblastoma.^126^ The role of S-palmitoylation in supporting cellular glycolysis has also been found in hepatocellular carcinoma. In contrast, the S-palmitoylation of hexokinase 1 (HK1) occurs in hepatic stellate cells, followed by proper localisation to the PM. In the presence of TSG101, HK1 is secreted extracellularly via vesicles formed by PM budding and further taken up by hepatocellular carcinoma cells to accelerate hepatocellular carcinoma progression by promoting tumour cell glycolysis.^127^

Tumour suppression by protein S-palmitoylation can be achieved by promoting the activation of tumour suppressor signalling pathways or blocking the activation of oncogene signalling pathways. S-palmitoylation of melanocortin-1 receptor (MC1R) in melanoma is a classic example of tumour suppression by activation of tumour suppressor signals. Activation of MC1R by α-MSH reduces the damage caused by ultraviolet irradiation by stimulating cAMP signalling and melanin production, but MC1R function is impaired in individuals with the MC1R RHC variant.^128^ It has been found that the S-palmitoylation of MC1R exerts a protective effect on melanomagenesis, and the S-palmitoylation of the MC1R RHC variant by DHHC13 can restore its function and activate MC1R signalling, thereby promoting pigmentation and controlling melanomagenesis.^128^ Subsequent studies further revealed that APT2 is responsible for MC1R depalmitoylation, and the maintenance of MC1R S-palmitoylation with the APT2 inhibitor ML349 effectively enhanced its downstream signalling and inhibited melanomagenesis.^129^ In addition, another study tried to enhance the interaction between DHHC13 and MC1R by regulating the activation of DHHC13. They found that AMPK could phosphorylate DHHC13 at S208 to activate DHHC13, so targeting AMPK is also an effective strategy to promote MC1R S-palmitoylation.^130^

Blocking the activation of tumour-promoting signalling pathways by enhancing the degradation of protumor factors is another way for S-palmitoylation to exert its antitumour effect. In breast cancer, DHHC22 is abnormally downregulated. DHHC22 mediates the S-palmitoylation of mTOR and reduces its stability in a time-dependent manner, thereby disturbing the PI3K/Akt/mTOR signalling pathway, inhibiting breast cancer cell proliferation and decreasing its resistance to neratini.^131^ Therefore, targeting DHHC22 is a powerful strategy for the treatment of breast cancer. In addition, S-palmitoylation can also block the activation of tumour-promoting signalling pathways by restricting the localisation of tumour-associated proteins. The oncogenic FLT3 mutation FLT3-ITD is one of the most common genetic changes in AML, which puts patients at higher risk of relapse and poor prognosis. The S-palmitoylation of FLT3-ITD maintains it localisation at the ER, while disruption of FLT3-ITD S-palmitoylation by palmitoylation site C563S mutation leads to its translocation to the PM, which promotes the activation of Akt and ERK and ultimately leads to the proliferation of leukaemia cells and the progression of leukaemia.^93^ In the majority of tumours where S-palmitoylation exerts an antitumour effect on associated proteins, this process is typically hindered. Therefore, enhancing S-palmitoylation of these tumour-associated proteins, such as the activation of the associated PAT enzymes or inhibition of depalmitoylases, represents a promising therapeutic strategy.

#### Neurodegenerative diseases

Protein S-palmitoylation plays a regulatory role in the brain, and dysregulation of protein S-palmitoylation has been found to be closely related to a variety of neurodegenerative diseases.^132^ Using Parkinson’s disease (PD) as an example, we will briefly discuss how aberrant S-palmitoylation regulation contributes to the aggregation of nonfunctional proteins, affecting the pathogenesis and progression of PD. An analysis of the palmitoyl proteome in the cerebral cortex of PD patients found that compared to controls, altered palmitoylation resulted in at least 150 proteins, many of which could interact with α-synuclein, LRRK2, DJ-1 and other proteins closely linked to the pathogenesis of PD, suggesting that abnormal S-palmitoylation regulation is a ubiquitous phenomenon in PD.^133^ For example, α-synuclein cannot be palmitoylated due to the lack of cysteine, but hyperphosphorylation leads to its misfolding and aggregation.^2^ Moreover, pathological α-synuclein accelerates the palmitate turnover of membrane vesicle-trafficking-related protein MAP6 and thus disrupts the normal association of MAP6 with vesicles. Enhancement of MAP6 S-palmitoylation by inhibition of APT1 may alleviate α-synuclein phosphorylation and neurotoxicity.^134^ In addition, the vesicle-trafficking protein syt11 can be palmitoylated at Cys39 and Cys40 to localise on the intracellular membranes instead of being degraded in lysosomes, and this modification leads to enhanced binding of α-synuclein to the intracellular membranes and the abnormal aggregation of α-synuclein in PD neurons,^135^ suggesting that the intervention of S-palmitoylation of proteins involved in the abnormal aggregation of α-synuclein may be a feasible measure for the treatment of PD.

#### Cardiovascular diseases

Studies have shown that S-palmitoylation can influence the function of cardiac myocytes by regulating ion channels and signal transduction.^136^ The important role of S-palmitoylation regulation in ion channel homeostasis and excitation-contraction coupling of cardiac myocytes has been discussed in detail in previous reviews.^136,137^ Using the S-palmitoylation of voltage-gated sodium channels (NaVs) as an example, we will briefly discuss its influence on cardiac electrical activity and its implications in heart diseases, highlighting the role of S-palmitoylation in maintaining cardiac homeostasis. Nav tightly controls the generation and propagation of sodium-dependent action potentials.^136^ As a heterotrimeric protein, S-palmitoylation often occurs in the early stages of Nav channel biosynthesis and induces different outcomes by palmitoylating cysteine sites in different regions.^136^ For example, the four palmitoylation sites of Nav1.5 are located between structural domains II and III, and S-palmitoylation may modulate the movement of the domain III voltage sensor and channel availability.^138,139^ Increased Nav1.5 S-palmitoylation induces enhanced cardiac excitability,^138^ which may be a potential mechanism of arrhythmia.

#### Inflammatory and autoimmune diseases

S**-**palmitoylation is implicated in the pathogenesis of several inflammatory and autoimmune diseases, such as inflammatory bowel disease, psoriasis, nonalcoholic steatohepatitis, and systemic lupus erythematosus.^86,117,140,141^ For instance, in colitis, the DHHC7 and APT2-mediated palmitoylation-depalmitoylation cycle of STAT3 enhances its activation, which in turn promotes TH17 cell differentiation to aggravate colitis.^117^ The excessive activation of TH17 cells is also linked with a variety of inflammatory and autoimmune diseases.^142^ Thus, targeting S-palmitoylation regulation of STAT3 to inhibit TH17 cells serves as a potential therapy for the treatment of these diseases. Moreover, in addition to regulating protein S-palmitoylation in TH17 cells, the pro-inflammatory activity of macrophages is also a potential target for intervention in colitis. After being palmitoylated in a FASN-dependent manner, Akt in colonic lamina propria mononuclear cells is recruited and localised to the PM and subsequently phosphorylated, which further activates downstream MAPK signalling to activate the pro-inflammatory effect of macrophages.^143^ Metformin, a traditional hypoglycaemic drug, disturbs the S-palmitoylation of Akt by inhibiting FASN, thereby suppressing pro-inflammatory responses.^143^ In addition, it was found that DHHC5-mediated S-palmitoylation of NOD1/2 is indispensable for their proper membrane recruitment and immune signalling, as defective S-palmitoylation of NOD1/2 was found to be associated with NOD-driven inflammatory diseases.^144^ Therefore, it is possible to balance immune activation by regulating each link of the protein S-palmitoylation in various immune cells involved in the inflammatory response and thus alleviate inflammatory and autoimmune diseases.

#### Infectious diseases

Emerging evidence has revealed the inextricable link between protein S-palmitoylation and infectious diseases, and a previous review has summarised the function of S-palmitoylation on key molecules of the innate immune response in infectious diseases.^10^ Using the S-palmitoylation regulation of SARS-CoV-2 proteins as an example, we will briefly explore its role in the viral infection process. SARS-CoV-2 relies on spike proteins to target cell binding and viral fusion. During viral infection, DHHC20 and DHHC9 were found to be responsible for S-palmitoylation of spike proteins in the ER and Golgi, which is followed by viral budding to mediate viral fusion and infection of host cells.^34,145^ Another study discovered that DHHC2, 3, 4, 5, 8, 9, 11, 14, 16, 19 and 20 all promoted the palmitoylation of the spike protein, and the palmitoylated spike protein promoted viral infection of host cells by mediating syncytium formation and the entry of SARS-CoV-2 pseudovirus particles into host cells.^146^ In addition, ACE2, the cell surface receptor interacting with the viral spike protein, is also regulated by DHHC3 and APT1 for its PM targeting and extracellular vesicle secretion.^147^ Therefore, intervention with the viral spike protein or ACE2 S-palmitoylation to block their interaction could inhibit viral fusion and infection to prevent and treat SARS-CoV-2 infection.

### Therapeutic targets and clinical research progress

Various agents targeting S-palmitoylation have been evaluated in preclinical studies (Table 1). However, these agents failed to enter clinical trials. In cancer research, strategies to inhibit S-palmitoylation in tumour-bearing animal models typically involve administering 2-BP or knocking down the associated DHHC-PATs. For example, inhibition of PD-L1 S-palmitoylation with 2-BP activates antitumour immunity in MC38 tumour-bearing mice.^98^ Nevertheless, the broad spectrum and toxicity of 2-BP are serious shortcomings that limit its wider application. Consequently, Han Yao and his team developed a competitive peptide inhibitor derived from the palmitoylation motif of PD-L1. This selective inhibitor was found to effectively downregulate PD-L1 expression.^98^ Interestingly, researchers have found that some popular clinical drugs can also inhibit protein S-palmitoylation. For example, the classical antimalarial drug artemisinin has been discovered to covalently bind DHHC6 and thus disturb its catalytic function, resulting in reduced S-palmitoylation of N-Ras, which could impair its downstream oncogenic signalling and thereby exert a potential anticancer effect by inhibiting protein S-palmitoylation.^148^ In addition, local anaesthetics, such as proparacaine, have been shown to decrease GP130 S-palmitoylation levels by suppressing DHHC15 transcripts. This action disrupts the IL-6/STAT3 signalling pathway and ultimately inhibits the growth and self-renewal of glioblastoma stem cells.^149^ In preclinical studies of other diseases, targeting S-palmitoylation remains a promising strategy. In CMA-treated mice, a small-molecule compound known as H-151 was identified to suppress STING palmitoylation, subsequently reducing systemic cytokine responses.^150^ The lipid peroxidation product 4-HNE is usually increased during viral infection. Interestingly, STING palmitoylation could be inhibited by 4-HNE, which induces carbonylation of STING at Cys88, indicating a new therapeutic target for the treatment of diseases with improper STING activation.^151^

**Table 1:** Summary of common inhibitors targeting protein lipidation

Lipidation	Inhibitors	Mechanism	Latest developmental stage	Effects or applications	Ref.
S-palmitoylation	2-bromopalmitate	Irreversible pan-depalmitoylation agent	In vitro and in vivo study	Inhibit tumour cell proliferation and tumour growth	^98^
	Metformin	Inhibition of fatty acid synthase-dependent palmitoylation	In vitro and in vivo study	Anti-inflammatory role in macrophages	^143^
	Artemisinin	Covalently binds and inhibits DHHC6	In vitro study	/	^148^
	Prilocaine	Weaken DHHC15 transcripts	In vitro and in vivo study	Inhibit tumour cell proliferation, self-renewal and tumour growth	^149^
	Lidocaine	Weaken DHHC15 transcripts	In vitro and in vivo study	Inhibit tumour cell proliferation, self-renewal and tumour growth	^149^
	Procaine	Weaken DHHC15 transcripts	In vitro and in vivo study	Inhibit tumour cell proliferation, self-renewal and tumour growth	^149^
	Ropivacaine	Weaken DHHC15 transcripts	In vitro and in vivo study	Inhibit tumour cell proliferation, self-renewal and tumour growth	^149^
	Palmostatin B	Broad-spectrum inhibitor of APT1/2, PPT1, ABHD17A/B/C as well as other lipid-processing serine hydrolases	In vitro study	Suppress cell viability in N-Ras mutant melanoma cells	^451^
	Palmostatin M	Broad-spectrum inhibitor of APT1/2, PPT1, ABHD17A/B/C as well as other lipid-processing serine hydrolases	In vitro study	Inhibit Legionella and Mycobacterium species growth	^452^
	ML211	Inhibition of APT1, APT2, ABHD11 and PPT1	In vitro study	/	^9^
	H-151	Occupy the palmitoylation site	In vitro and in vivo study	Reduce cytokine responses	^150^
	HCQ	Inhibition of PPT1	In vivo study	Inhibit tumour growth of HCC in xenograft model	^83^
	ML348	Inhibition of APT1	In vitro and in vivo study	Reverse neuropathology, locomotor deficits, and anxio-depressive behaviours; deteriorate myocardial pyroptosis, infarct size and cardiac function in AML mice	^453,454^
	ML349	Inhibition of APT2	In vitro and in vivo study	Repress UVB-induced melanomagenesis	^129^
	ABD957	Inhibition of ABHD17	In vitro study	Inhibit N-Ras-mutant AML cell growth	^78^
N-myristoylation	2-hydroxymyristic acid	Inhibition of NMTs	In vitro study	Inhibit viral multiplication	^455,456^
	D-NMAPPD (B13)	Inhibition of NMT1	In vitro and in vivo study	Inhibit cell-cycle progression and growth of xenograft tumours	^199^
	LCL204	Inhibition of NMT1	In vitro study	/	^199^
	Tris-DBA palladium	Inhibition of NMT1	In vitro and in vivo study	Inhibit melanoma cell proliferation and growth of tumours	^225^
	IMP-366 (DDD85646)	Inhibition of NMT1 and NMT2	In vitro and in vivo study	Inhibit *Trypanosoma cruzi*	^457^
	IMP-1088	Inhibition of NMT1 and NMT2	In vitro study	Inhibit the replication of rhinovirus, poliovirus, foot-and-mouth disease virus, and vaccinia virus	^233^
	IMP-1002	Inhibition of *Plasmodium falciparum* NMT	In vitro study	Disrupt *Plasmodium falciparum* development and growth	^458^
	IMP-105	Inhibition of Leishmania NMT	In vitro study	Anti-Leishmanial activity	^459^
	RO-09-4879	Inhibition of Candida albicans NMT	In vitro study	Antifungal activity against *Candida albicans*	^460^
	PCLX-001	Inhibition of NMT1 and NMT2	phase I clinical trial	Test in B-cell non-hodgkin lymphoma and advanced solid malignancies	^461^
	Asciminib	targeting the myristoyl pocket of the BCR-ABL1 tyrosine kinase	FDA approval	Treatment of adult patients with Ph+ CML-CP, previously treated with ≥ 2 TKIs, and Ph+ CML-CP with the T315I mutation	^232^
S-prenylation	Statin	Inhibition of FPP and GGPP biosynthesis	Phase II clinical trial	Test in HGPS	/
	Bisphosphonates	Inhibition of the FPPS activity and the synthesis of FPP and GGPP	Phase II clinical trial	Test in Progeria	/
	FPP analogues	Inhibition of FTase	In vitro study	/	^328^
	BMS-184467	Inhibition of FTase	In vitro study	Inhibit the growth of ras transformed cells	^329^
	BMS-185878	Inhibition of FTase	In vitro study	Inhibit the growth of ras transformed cells	^329^
	BMS-214662	Inhibition of FTase	Phase I clinical trial	Test in solid cancer and haematologic disease	^462,463^
	tipifarnib	Inhibition of FTase	Phase III clinical trial	Test in solid cancer, haematologic disease	^464,465^
	lonafarnib	Inhibition of FTase	FDA approval	Treatment of HGPS and processing-deficient progeroid laminopathies	^466^
	GGTI-2418	Inhibition of GGTase-I	Phase I clinical trial	Test in advanced malignancies	^330^
S-prenylation	GGTI-2154	Inhibition of GGTase-I	In vitro and in vivo study	Inhibit tumour growth of H-Ras transgenic mice	^467^
	FGTI-2734	Inhibition of FTase and GGTase-I	In vitro and in vivo study	Inhibit tumour cell proliferation and tumour growth; suppress mast cell-dependent anaphylaxis	^348,349^
	L-778,123	Inhibition of FTase and GGTase-I	Phase I clinical trial	Test in advanced solid cancers	^351,467^
	NSC1011	Inhibition of RCE1	In vitro study	/	^468^
	UCM-1336	Inhibition of ICMT	In vitro and in vivo study	Induce cell death in Ras-mutated tumour cell lines and inhibiting tumour growth	^353,469^
	Cysmethynil	Inhibition of ICMT	In vitro and in vivo study	Induce cell death in tumour cells and inhibiting tumour growth	^470^
	Compound 8.12	Inhibition of ICMT	In vitro and in vivo study	Induce cell death in tumour cells and inhibiting tumour growth	^358^
	C75	Inhibition of ICMT	In vitro and in vivo study	Delay ageing and promote the proliferation of HGPS Cells	^360^
GPI anchor	SHAM	Inhibition of protozoan de-N-acetylase in the GPI pathway	In vitro study	Suppress *P. falciparum* 3D7 growth at trophozoite stages	^471^
	BIQ	Inhibition of fungal Gwt1 in the GPI pathway	In vitro study	Reduce *C. albicans* cell adherence	^472^
	Gepinacin	Inhibition of fungal Gwt1 in the GPI pathway	In vitro study	Antifungal activity against *C. albicans*	^473^
	G365	Inhibition of fungal Gwt1 in the GPI pathway	In vitro study	Antifungal potency against *C. albicans*	^474^
	G884	Inhibition of fungal Gwt1 in the GPI pathway	In vitro study	Antifungal potency against *C. albicans* and others	^474^
	Compound A1	Inhibition of fungal Gwt1 in the GPI pathway	In vitro and in vivo study	Antifungal potency against drug-resistant *C. auris* and *C. albicans*	^427^
	APX001	Inhibition of fungal Gwt1 in the GPI pathway	Phase II clinical trial	Test in candidemia and other invasive fungal infections	^429,430^
	M743	Inhibition of fungal MCD4 in the GPI pathway	In vitro study	Antifungal potency against *C. albicans* and others	^474^
	M720	Inhibition of fungal MCD4 in the GPI pathway	In vitro study	Antifungal potency against *C. albicans* and others	^474^
Cholesterylation	CID 5717	Inhibition of the cholesterol-dependent autocleavage of Hh protein	In vitro study	/	^449^
	CID 72303	Inhibition of the cholesterol-dependent autocleavage of Hh protein	In vitro study	/	^449^

*2-BP* 2-bromopalmitate, *UVB* ultraviolet B, *HCC* hepatocellular carcinoma, *CML-CP* chronic myelogenous leukaemia in chronic phase, *TKI* tyrosine kinase inhibitor, *AML* acute myelocytic leukaemia, *HGPS* Hutchinson-Gilford progeria syndrome

### N-myristoylation

#### Regulatory enzymes and catalytic mechanism

NMTs belong to the GCN5-related N-acetyltransferase superfamily.^152^ While lower eukaryotes typically have a single NMT gene, higher organisms possess both NMT1 and NMT2, which share nearly 77% peptide sequence identity in *Homo sapiens*.^153^ To some extent, NMT1 and NMT2 exhibit partial substrate overlap and functional redundancy. This is evident as depleting either isozyme can trigger apoptosis.^154^ However, the physiological complexity predicts that the two isozymes also have functional specificity and substrate preference, which could be reflected in the different roles of NMT1 and NMT2 during embryogenesis and tumour cell proliferation, with NMT1 playing a critical role in these physiological and pathological processes.^154–156^ During apoptosis, caspases cleave NMTs, causing NMT1 to move from the PM to the cytosol and relocate NMT2 in the opposite direction,^157^ and this positional change may influence its substrate specificity.

**Fig. 5:**
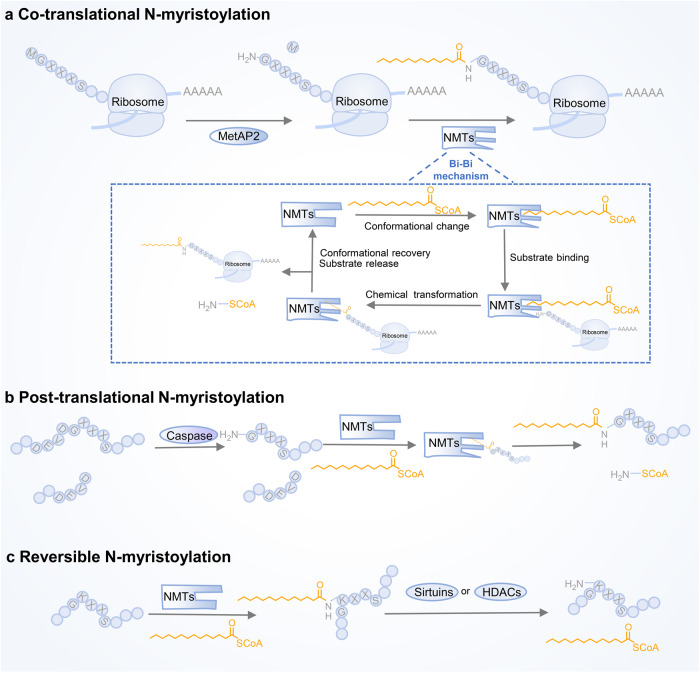
The catalytic mechanism and process of protein N-myristoylation. **a** Cotranslational myristoylation usually occurs on the glycine at the N-terminal end of nascent proteins after the methionine initiator has been removed by MetAP2. The catalytic mechanism follows the Bi–Bi mechanism. Conformational changes were induced after NMTs bound to the fatty acid chain of the myristoyl-CoA binding site. Then, the substrate binds to the NMTs and produces myristoylpeptide via a myristoyl transfer reaction. Finally, the NMTs release the myristoylpeptide and restore its conformation. **b** Posttranslational protein N-myristoylation often occurs during apoptosis. After the internal glycine of the substrate protein is exposed by caspase cleavage, NMTs catalyse the attachment of myristic acid to the glycine residue of the substrate. **c** Reversible protein N-myrisotylation occurs on the Nε-side chain of lysine residues instead of glycine residues, which is reversed by sirtuins or HDACs

Through kinetic and X-ray crystallographic research, the molecular mechanisms underlying NMT catalysis have been revealed.^13,158–161^ Briefly, both cotranslational and posttranslational N-myristoylation obey a well-organised Bi−Bi mechanism involving a direct nucleophilic addition-elimination reaction (Fig. 5). First, NMT binds the fatty acid chain of myristoyl-CoA to form the myristoyl-CoA-NMT complex and induces conformational changes to expose the substrate-binding site. Subsequently, the enzyme-substrate complex is converted to the enzyme product complex via a myristoyl transfer reaction to release CoA and myristoylpeptide. Finally, NMT returns to its original conformation, hiding its substrate-binding pocket.

Cotranslational myristoylation usually occurs on the glycine at the N-terminal end of nascent proteins after the methionine initiator has been removed by MetAP2, and the N-terminal region of NMTs is necessary for isozyme-specific binding to the ribosome.^14,159,162,163^ The majority of myristoylated proteins responsible for basal cell function undergo cotranslational modifications, whereas during apoptosis, a portion of myristoylated proteins undergo posttranslational modifications when the internal glycine is exposed by caspase cleavage.^164^ Myristoylation usually occurs at the protein NH2-terminal glycine and is considered irreversible. However, research has found that myristate can covalently attach to some proteins that lack NH2-terminal glycine. Further studies revealed that the Nε-side chain on Lys residues undergoes myristoylation, and NMTs are the enzymes responsible for catalysing the myristoylation of lysine.^16,165^ Intriguingly, fatty acyl groups on Nε-modified lysine residues can be removed by sirtuins and HDACs, suggesting that in addition to palmitoylation, lysine myristoylation is also dynamic and reversible^17,166,167^ (Fig. 5). As a deacetylase, SIRT6 not only hydrolyses lysine myristoylation of proteins such as TNF-α, but its activity as a demyristoylation enzyme is much higher than its deacetylation activity, indicating that the regulation of intracellular deacylation activity is extremely delicate and complex.^167^ Lysine myristoylation of gravin-α is needed for GPCR signalling, and demyristoylation of gravin-α by HDAC11 inhibits this pathway, suggesting that reversible lysine myristoylation is involved in the delicate regulation of GPCR signalling.^168^

### Physiological function

#### Intracellular trafficking and protein-lipid interactions

Similar to S-palmitoylation, attaching myristoyl acids to proteins enhances their ability for intracellular trafficking and increases membrane-binding affinity. Nevertheless, this increased affinity by N-myristoylation is essential but not sufficient for protein anchoring to the membrane.^169,170^ Unlike S-palmitoylation, the myristoyl component of proteins undergoes more dynamic changes, as reflected by the fact that the myristoyl moiety can either be hidden in the hydrophobic pocket of the protein or exposed for membrane binding, and it was previously speculated that these two conformational changes are regulated under the assistance of several secondary signals, termed the myristoyl switch.^171–174^ The first type of myristoyl switch is the myristoyl-electrostatic switch. The myristoyl component works in synergy with electrostatic interactions, especially between the protein’s positively charged effector domain and phospholipids, facilitating protein binding to the membrane.^174,175^ Serines on such domains are highly susceptible to phosphorylation thereby altering electrostatic attraction. For instance, myristoylated MARCKS would detach from the membrane and translocate to the cytosol when phosphorylated by protein kinase C, while it could reattach to the membrane once it was dephosphorylated by phosphatases.^176,177^ Interestingly, even without assisting motifs, a portion of PKA-C can focus PKA activity once its electrically neutral N-terminus undergoes myristoylation, indicating that myristoylation alone is sufficient for this portion of PKA-C to interact with the membrane under certain circumstances.^178^ The second type of myristoyl switch is the myristoyl-ligand switch. A typical example of being subject to such a switch is the retinal calcium-binding protein Recoverin, whose myristoyl moiety needs to be exposed upon Ca (2 + )-binding sites of N-terminal binding to calcium, and this binding induces not only the myristoyl moiety but also many hydrophobic residues for membrane localisation.^179,180^ The third type is the myristoyl-palmitoyl switch. Here, in addition to N-myristoylation, proteins undergo an additional modification, usually S-palmitoylation, to ensure robust membrane binding. Proteins that require these two lipidations for anchoring to the membrane include FCaBP of *Trypanosoma cruzi*,^181^ H-Ras,^182^ FRS2α,^183^ RNF11^184^ and others. Intriguingly, S-palmitoylation alters the localisation preferences of some myristoylated proteins. For example, N -myristoylated but not palmitoylated Gαi1 predominantly bound to ordered lipid microstructural domains with a net negative charge. In contrast, Gαi1 containing both FAs preferentially interacted with nonnegatively charged raft-like lamellar membranes.^185^

#### Protein stability and degradation

Like S-palmitoylation, N-myristoylation plays a role in controlling protein stability. Quite a few proteins can be altered not only in their intracellular localisation but also in their fate of being degraded after undergoing myristoylation. For instance, myristoylation not only facilitates FSP1 membrane translocation but also promotes its stability by evading the proteasome degradation pathway.^186^ Another study discovered that myristoylation also protects VILIP3 from lysosomal pathway-mediated degradation.^187^ In some cases, myristoylation only enhances protein stability without causing significant changes in membrane binding. The presence or absence of myristoylation only affects the thermal stability of calcineurin but not its binding to phospholipid monolayers.^188^ As early as 1986, the presence of N-degrons that exert degradation signals at the N-terminus of proteins was discovered,^189^ and theories relevant to N-degron degradation have been continuously updated.^190^ A subsequent study revealed that protein myristoylation prevented the N-terminal glycine from being exposed, thereby eclipsing the selective proteasomal degradation mediated by Cul2^ZYG11B^ and Cul2^ZER1^^191^ However, myristoylation does not invariably shield proteins from degradation. For instance, compared to myristoylated c-Src, nonmyristoylated c-Src resists proteasome-mediated degradation, which is achieved by reducing its association with the ubiquitin E3 ligase Cbl.^192^ These studies demonstrated that the regulation of the conformation changes of N-terminal sites by myristoylation is sophisticated and thus delicately regulates protein degradation.

#### Protein–protein interactions

Apart from modulating intracellular trafficking, membrane attachment and protein stability, myristoylation also caters to cellular and viral needs via its interactions with other proteins. A prime example of how myristoylation participates in protein–protein interactions is Src.^193^ p60v-src of Rous sarcoma virus is essential for the transformation activity of the virus, and studies have shown that this transforming activity is largely dependent on the interaction of p60v-src with the membrane. Specifically, this interaction relies on the binding of myristoylated p60v-src to receptor SLC25A5.^193,194^ Another well-studied example illustrating the impact of myristoylation on protein–protein interactions is Gag. As a major coordinator of the assembly process of many retroviruses, its N-terminal matrix domain is myristoylated to target the PM and anchor.^195–197^ Yiping Zhu et al. found that the host factor HO-2 could impede HIV replication by specifically binding to the myristic acid portion of Gag; such binding requires the myristoylation of HO-2, and blocking HO-2 myristoylation would lead to an increase in viral replication.^198^

#### Signal transduction

**Fig. 6:**
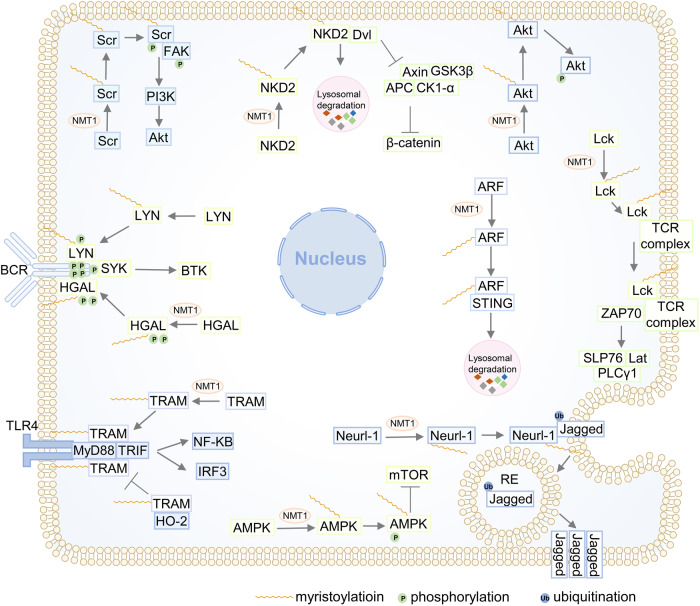
N-myristoylation is involved in the regulation of multiple cellular signal transduction pathways. We briefly summarised several well-established signalling pathways that are under the control of N-myristoylation, including the Scr-meditating oncogenetic pathway, Wnt pathway, Akt pathway, STING-autophagy pathway, Notch signalling pathway, TCR activation signalling pathway, TLR4 inflammatory responses, AMPK signalling pathway and B-cell receptor pathway

N-myristoylation plays a pivotal role in cellular signalling pathways, primarily by modifying protein intracellular trafficking, membrane association, and protein–protein interactions, among other functions. Accumulating evidence suggests that myristoylation has a substantial impact on various signalling pathways that directly or indirectly regulate cancer, metabolism, immunity and others, such as the Src signalling pathway,^199^ AMPK signalling pathway,^200^ Wnt signalling pathway,^201^ PI3K/Akt signalling pathway,^202^ Notch signalling pathway,^203^ the LPS-induced TLR4 inflammatory response,^204^ the cGAS-STING signalling pathway,^205^ the B-cell receptor pathway^206^ and pathways involved in T-cell development^207^ (Fig. 6). For instance, AMPK, an αβγ heterotrimer, is a crucial regulator of cellular energy homeostasis. It plays a vital role in cellular energy sensing and bioenergetics and is linked with the regulation and progression of numerous diseases.^208^ In rheumatoid arthritis, due to defective NMT1 function in T cells, the inability of AMPK to be myristoylated impedes its lysosomal recruitment and activation, which leads to the overactivation of the mTORC1 pathway and promotes T-cell differentiation into pro-inflammatory TH1 and TH17 helper T cells, resulting in severe synovial tissue inflammation.^209^ Recently, it has been found that myristoylation is involved in the regulation of the STING pathway mainly through the modulation of ARF1, which is a major regulator of STING membrane trafficking, and its function is dependent on N-myristoylation.^205^ Therefore, the myristoylation of ARF1 is an important target for balancing STING-dependent autophagy and IFN responses to promote immune homeostasis by regulating STING pathway activation. For Notch signalling, myristoylation of Neurl-1 is indispensable for endocytosis and relocalization of the PM of the Notch ligand jagged 1.^203^

### Pathological implication

#### Cancer

Numerous studies indicate that while NMT1 is crucial for organismal growth and development, it is also an oncogenic protein that is aberrantly expressed in many human cancers, suggesting that NMT1-mediated protein myristoylation is a potential mechanism of tumorigenesis and development. In lung cancer, EZH2, which undergoes myristoylation, is notably highly expressed. The resulting hydrophobic interaction can drive and stabilise liquid-liquid phase separation. Myristoylated EZH2 binds STAT3 to recruit it to phase-separated droplets, thereby overactivating the STAT3 pathway to promote lung cancer progression.^210^ In bladder cancer, upregulated NMT1 causes the lysosomal anchoring protein LAMTOR1 to be myristoylated at Gly2, enhancing its protein stability and lysosomal localisation, which leads to the activation of the mTORC1 pathway and mediates bladder cancer progression.^211^ In HCC, upregulated NMT1-mediated myristoylation of the VILIP3 protein enhances its stability and subsequent NFκB/Bcl-2 signalling, thereby contributing to the progression of HCC.^187^ In ovarian cancer, upregulated ACSL1 is involved in metabolic reprogramming, which enhances fatty acid beta-oxidation in tumour cells by promoting the myristoylation of a series of substrate proteins, such as AMPKβ, Src, and FSP1, thereby regulating the progression and metastasis of ovarian cancer.^186,212^ In addition, myristoylation also plays an essential regulatory role in certain tumours driven by specific oncogenes, such as EGFR-dependent tumours. For EGFR, translocation from the Golgi to the PM is a prerequisite for its pro-oncogenic role, and this process requires the recognition and binding of EGFR by myristoylated ARF6, which facilitates its budding from the Golgi and its translocation in the GTP-bound form.^213^

#### Metabolic disorders

Myristoylation dysregulation is a frequent occurrence in metabolic disorders. A 2-fold increase in NMT activity was observed in a rat model of insulin-dependent diabetes mellitus induced by STZ compared to controls, whereas a nearly 5-fold decrease in NMT activity was observed in a rat model of non-insulin-dependent diabetes mellitus compared to controls.^214,215^ NMT activity appears to be negatively correlated with plasma insulin levels.^215^ Furthermore, saturated FAs were found to enhance the membrane localisation of c-Src by promoting its myristoylation, therefore causing insulin resistance through JNK pathway activation, while unsaturated FAs had the opposite effect.^216^ Furthermore, obesity, another prevalent metabolic disorder, often accompanies the abnormal regulation of myristoylation in several pathogenesis-linked proteins. Similar to type 2 diabetes, obesity is affected by saturated FA-mediated activation of the JNK pathway.^216^ In addition, abnormal regulation of leptin signalling is quite common in the development of obesity, and studies have shown that it is myristoylated Akt but not nonmyristoylated Akt that induces increased leptin levels in 3T3-L1 adipocytes,^217^ which indicates that targeting Akt myristoylation may present a promising way to improve obesity.

#### Autoimmune disorders

In rheumatoid arthritis (RA), aberrant regulation of AMPK myristoylation leads to abnormal CD4 T-cell differentiation and triggers inflammation. Specifically, low expression of NMT1 inhibited myristoylation-dependent AMPK lysosomal trafficking and activation, which resulted in the overactivation of mTORC1 signalling in CD4 T cells and contributed to the differentiation of CD4 T cells into pro-inflammatory Th1 and Th17 cells^209,218^ Moreover, a strong anti-inflammatory effect could be achieved by overexpressing NMT1 in RA T cells with low NMT1 expression.

#### Infectious diseases

Research indicates that the survival and invasion of several pathogens, including viruses and parasites, hinge on protein myristoylation. In the absence of NMTs, several viruses exploit the host’s NMTs to myristoylate essential assembly proteins, facilitating its assembly and replication. A typical example is HIV. Myristoylated Gag guides the virus and anchors it to the PM, demonstrating that this structural protein has a critical effect on viral assembly.^195^ In the absence of Gag or when the myristoylation site is mutated, HIV-1 RNA is highly dynamic and is unable to either anchor to the membrane or complete the assembly of virions correctly.^197^ As a myristate-binding protein, HO-2 of the host blocks HIV membrane localisation by selectively binding to the myristate moiety of Gag, thereby impeding viral assembly and replication.^198^ The consequence of HIV infection is that it causes the progressive loss of CD4 T cells and widespread immune abnormalities in the host, and the myristoyl protein Nef has been found to have a pivotal function in compromising host immunity.^219^ Binding of Nef to the PM through myristoylation induces rapid internalisation of CD4 on the surface and MHC-1 of T cells, leading to its degradation in lysosomes.^220,221^ In addition, Nef negatively regulates phagocytosis in macrophages. Through interaction with AP-1, myristoylated Nef disrupts the membrane delivery of VAMP3- and TNFα positive endosome compartments and impairs optimal phagosome formation, thereby inhibiting macrophage phagocytosis.^222^ Overall, myristoylation is involved in multiple aspects of HIV infection of the host, supporting viral replication and immune evasion by regulating the membrane binding and protein–protein interactions of multiple proteins.

### Therapeutic targets and clinical research progress

Growing evidence strongly supports the development of therapeutic targets against NMT activity and myristoylation. Currently, the development of related drugs mainly focuses on antitumour and anti-infection activities (Tables 1 and 2). For tumours with elevated NMT expression and activity, several compounds are available as NMT inhibitors. The usage of myristoyl-CoA analogues to inhibit NMT dates back as far as 1990, when researchers compared the inhibitory effects of three myristic acid derivatives: 2-fluoromyristic acid, 2-bromomyristic acid and 2-hydroxymyristic acid.^223^ All three compounds exerted weak inhibitory effects on NMT. Subsequent synthesis of these myristic acid derivatives into 2-substituted acyl-CoA analogues by acyl-CoA synthase significantly enhanced the inhibitory effect on NMT.^223^ Investigating how structural changes in acyl-CoA derivatives alter the binding of protein substrates will provide remarkably useful information for the design of potent antitumour compounds targeting NMT. Another myristoyl-CoA analogue, B13, also known as D-NMAPPD, potently inhibited NMT1 enzymatic activity against prostate cancer cells.^199^ By competing with the myristoyl-CoA binding site of NMT1, B13 impaired the myristoylation of Src, leading to the blocking of relevant oncogenic signalling and thereby promoting the antitumour effect. Furthermore, by optimising the structure of B13, the derivative LCL204 exhibited a lower IC50 for NMT1 enzymatic activity.^199^ However, a later study obtained an inconsistent conclusion that B13 was not an NMT inhibitor, as no changes in N-myristoylation in c-Src were observed when MDA-MB-231 or HeLa cells were treated with B13 at 30 μM.^224^ Tris dipalladium (Tris DBA) is another NMT inhibitor with debatable efficacy. A previous study demonstrated that the expression of NMT1 at the mRNA and protein levels and cell proliferation were inhibited after Tris-DBA treatment of B16 and A375 cells.^225^ However, there is no direct evidence for the interaction of Tris DBA with NMT1 and its effect on protein myristoylation.^225^ Wouter W Kallemeijn et al. further discovered that Tris DBA causes cytotoxicity independent of NMT and myristoylation,^224^ suggesting that the characterisation of Tris DBA as an NMT1 inhibitor needs to be revisited. In addition to developing small-molecule compounds such as myristoyl-CoA analogue, targeting biological NMT inhibitors including NIP71 and HSC70 as well as enolase is also a promising approach. Studies initially found that NIP71 was negatively correlated with the expression of the NMT substrate pp60^Src^, and subsequently revealed that purified NIP71 and HSC70 could inhibit human NMT in a dose-dependent manner.^226,227^ Similarly, enolase was found to inhibit human NMT activity in a dose-dependent manner.^228^ The discovery of these biological NMT inhibitors has broadened the strategy of targeting NMT for the treatment of tumours, but more studies are needed to confirm the effectiveness of their application.

**Table 2:** Clinical trials of various diseases by targeting protein lipidation

Lipidation	Drug	Mechanism	Disease or patients	Treatment	Phase	NCT number
N-myristoylation	PCLX-001	Pan-NMT inhibitor	Relapsed/refractory B-cell non-Hodgkin lymphoma and advanced solid malignancies	Monotherapy	I	NCT04836195
	Asciminib (ABL001)	Target the myristoyl pocket of the BCR-ABL1 tyrosine kinase	Ph+ ALL or CML	Asciminib in combination with Dasatinib, Prednisone, and Blinatumomab	I	NCT03595917
			CML	Monotherapy of imatinib or combination with asciminib; combination of asciminib with TKI	II	NCT04216563
			CML	Continued treatment of asciminib	IV	NCT04877522
			Newly diagnosed CML-CP	Monotherapy or combination with one TKI	II	NCT05143840
			Newly diagnosed, previously untreated Ph+ CML-CP	Monotherapy of asciminib or nilotinib; or one selected TKI	III	NCT05456191 NCT04971226
			CML-CP previously treated with one prior ATP-binding site TKI	Monotherapy	II	NCT05384587
			Relapsed CML previously attempted to discontinue imatinib	Asciminib combination with imatinib	II/ III	NCT05413915 NCT04838041
			CML-CP previously treated with two or more TKIs	Monotherapy of asciminib; or best available treatment; or bosutinib	II/III	NCT04795427 NCT04948333 NCT03106779
			CML-CP without T315I mutation previously treated with two prior TKIs; CML-CP with T315I mutation with previously treated with at least prior TKIs	Monotherapy	III	NCT04666259
S-prenylation	Pravastatin	Inhibition of FPP and GGPP biosynthesis	HGPS	A combination of zoledronic acid and pravastatin	II	NCT00731016
	Zoledronic acid	Inhibition of the FPPS	Progeria; HGPS	A combination of pravastatin and lonafarnib and zoledronic acid	II	NCT00879034 NCT00916747
	Lonafarnib	FTase inhibitor	HDV infection	Monotherapy	II 11	NCT01495585
			HDV infection	Lonafarnib in combination with ritonavir	II/III	NCT02527707 NCT02511431 NCT05229991
			HDV infection	Lonafarnib With or Without Ritonavir	II	NCT02968641 NCT02430181
			HDV infection	A combination of Lonafarnib, ritonavir and peginterferon lambda-1a	II	NCT03600714 NCT05953545
	BMS-214662	FTase inhibitor	acute leukaemia, MDS, or CML	Monotherapy	I	NCT00006213
			Solid tumours	Monotherapy	I	NCT00005973 NCT00004877
			Advanced solid tumours	Monotherapy; BMS-214662 in combination with trastuzumab; BMS-214662 in combination with paclitaxel	I	NCT00004877 NCT00022529 NCT00006018
S-prenylation	Lonafarnib	FTase inhibitor	HGPS	monotherapy	II	NCT00425607
			Progeria	Lonafarnib in combination with and everolimus	I/II	NCT02579044
			MDS or CML	Monotherapy	III	NCT00109538
			Chronic or accelerated phase CML	Monotherapy; Lonafarnib in combination with Gleevec	I/II	NCT00038597 NCT00047502
			Metastatic breast cancer	Monotherapy	II	NCT00773474
			Advanced breast cancer	Lonafarnib in combination with Herceptin Plus Paclitaxel; anastrozole in combination with lonafarnib or monotherapy of anastrozole	II	NCT00068757 NCT00081510
			Recurrent or progressive brain tumours in children	Monotherapy	I	NCT00015899
			HNSCC	Monotherapy	I/II	NCT00038584 NCT00073450
			Advanced or recurrent HNSCC	Lonafarnib in combination with Fenretinide	I	NCT00102635
			Advanced ovarian carcinoma	Monotherapy	II	NCT00281515
			Glioblastoma multiforme	Lonafarnib in combination with temozolomide	I	NCT00102648
			Recurrent glioblastoma multiforme	Lonafarnib in combination with temozolomide	II	NCT00038493
			Recurrent primary supratentorial gliomas	A combination of lonafarnib and temozolomide	I	NCT00083096
			Grade 3 & 4 malignant gliomas	Lonafarnib in combination with Temodar	I	NCT00612651
			Metastatic colorectal cancer	Monotherapy or conventional surgery	I	NCT00005030
			Stage 3b or 4 NSCLC	Lonafarnib in combination with, paclitaxel and carboplatin	III	NCT00050336
			Advanced cancer of the urinary tract	Lonafarnib in combination with gemcitabine	II	NCT00006351
			Advanced malignancies	Lonafarnib in combination with docetaxel; Lonafarnib in combination with fluorouracil, and leucovorin	I	NCT00288444 NCT00003956
	Tipifarnib	FTase inhibitor	Leukaemia	Monotherapy	I/II	NCT00022451 NCT00004009 NCT02807272
			AML	Monotherapy; Tipifarnib in combination with chemotherapy;	I/II/III	NCT00124644 NCT00027872 NCT01361464 NCT00354146 NCT00093418 NCT00048503 NCT00093990 NCT00093470
			Advanced haematologic cancer	Monotherapy	I	NCT00005967
			AML	Tipifarnib in combination with Etoposide	I/II	NCT00112853 NCT00005989
			CML-CP	Tipifarnib in combination with Imatinib mesylate	I	NCT00040105
			Myeloproliferative disorders	Monotherapy	I/II	NCT02210858 NCT02779777
			Multiple myeloma	Monotherapy or Tipifarnib in combination with Bortezomib or PS-341	I/II	NCT00012350 NCT00972712 NCT00361088

*Ph* *+* Philadelphia chromosome positive, *ALL* acute lymphoblastic leukaemia, *CML-CP* chronic myelogenous leukaemia in chronic phase, *TKI* tyrosine kinase inhibitor, *HGPS* Hutchinson-Gilford progeria syndrome, *MDS* myelodysplastic syndrome, *HNSCC* head and neck squamous cell carcinomas, *NSCLC* non-small cell lung cancer, *HDV* hepatitis delta virus

To date, clinical studies of NMT inhibitors targeting tumours have focused on haematologic malignancy. For instance, a phase I clinical trial showed that asciminib, by binding to the myristoyl site of the BCR-ABL1 protein, effectively inactivates it, benefiting CML patients with TKI resistance or T315I mutations.^229^ In a phase III clinical study evaluating the major molecular remission (MMR) rate after treatment with asciminib or bosutinib in patients with CML-CP who were previously resistant/intolerant to at least two TKIs, the MMR of asciminib was significantly higher than that of bosutinib at week 24.^230^ Another clinical study at a more distant observation point (week 96) also discovered that the MMR of asciminib was superior to that of bosutinib.^231^ In 2021, asciminib received FDA approval for the treatment of adults with Ph+ CML in the chronic phase (Ph+ CML-CP), previously treated with ≥ 2 TKIs, and Ph+ CML-CP with the T315I mutation.^232^ These encouraging clinical data suggest that targeting protein myristoylation is highly promising, motivating researchers to explore further development of relevant drugs to expand their clinical applications in the future.

In addition to anti-tumours, studies targeting protein myristoylation for the development of anti-infective drugs are also in full swing. For instance, the recently identified compound IMP-1088 acts as a novel NMT inhibitor. It effectively hinders the assembly and replication of various viruses, including rhinoviruses and vaccinia virus, by blocking the myristoylation of their proteins,^233,234^ thus highlighting the potential of protein myristoylation as a drug target for a wide range of viral infections. In addition, selective inhibitors targeting NMT, including myristate analogues, myristoylpeptide derivatives, histidine analogues (peptidomimetics), aminobenzothiazoles, quinolines and benzofurans showed broad-spectrum antifungal activity.^235–237^ In addition, given the difference in NMT between humans and microorganisms, NMT is an attractive target for antiparasitic drugs, and several compounds that have demonstrated antiparasitic activity against *Trypanosoma brucei*, *Plasmodium falciparum* and *Plasmodium vivax*^238–241^ could be used in the development of anti-malaria and anti-Human African trypanosomiasis drugs. These findings further validate NMTs and myristoylation as exciting drug targets for the treatment of a myriad of diseases, and related drugs can be further optimised to become more effective and safer.

## S-prenylation

### Regulatory enzymes and catalytic mechanism

Currently, four known heterodimeric prenyltransferases are responsible for the prenylation of proteins: protein aryltransferase (FTase), protein gammaglutamyltransferase type I (GGTase-I), Rab geranylgeranyltransferase (GGTase-II) and protein gammaglutamyltransferase type III (GGTase-III).^21,242^ The first two prenyltransferases are responsible for adding a farnesyl or geranylgeranyl group to the C-terminus of the substrate protein. FTase and GGTase-I are both heterodimers and share the common α subunit FNTA or PTAR2, but their β subunit is different ((FNTB in FTase and PGGT1B in GGTase-I), which shapes their reactivity and substrate specificity.^243,244^ Structural analyses indicate that most prenylated proteins have a CAAX motif at their C-terminus, serving as the enzyme recognition site, where “C” denotes a cysteine residue, “A” usually indicates hydrophobic amino acids, and “X” denotes any amino acid that determines whether the protein is farnesylated or geranylgeranylated.^245^ Generally, farnesylation selectively occurs in proteins with alanine, methionine, serine, or glutamate at the “X” position, whereas fam prefers proteins with leucine, methionine, phenylalanine, isoleucine and valine at the “X” position.^246–248^ Despite the well-recognised substrate selectivity of these two prenyltransferases, some substrate proteins can be regulated by both FTase and GGTase-I. For example, an earlier study in yeast showed that Ras is a substrate for FTase, but overexpression of GGTase-I in an FTase-deficient yeast strain compensated for the farnesylation of Ras and rescued growth defects caused by FTase deficiency.^249^ In addition, the farnesylation of K-Ras could not be completely inhibited when using an FTase inhibitor, as it would be catalysed by GGPTase-I.^250^ To some extent, this cross-prenylation process adequately ensures that some genes, such as Ras perform physiological functions. However, for the RhoB protein, different farnesylation modifications yield different functions. The farnesylated form of the RhoB protein is mainly involved in cell growth, whereas the geranylgeranylated form of the RhoB protein mainly induces apoptosis.^251^ GGTase-II, the third protein prenyltransferase, comprises the α subunit PTAR3 and the β subunit RabGGTB. For GGTase-II, whose subunits consist of RabGGTA (PTAR3) and RabGGTB, the catalytic feature is that it can contribute to both monogeranylgeranylation of cysteine residues near the C-terminus of the substrate protein and dual geranylgeranylations of the two cysteine residues near the C-terminus. Specifically, monogeranylgeranylation occurs when the C-terminus of the substrate protein contains the CAAX or CXXX motif, while double geranylgeranylation occurs when the C-terminus of the substrate protein contains the CC, CXC, CCX, CCXX or CCXXX motif.^21^ Dual geranylgeranylation is not redundant, as the substrates of GGTase-II Rab proteins require dual geranylgeranylation to properly mediate vesicular trafficking.^252^ GGTase-III is a fourth protein prenyltransferase composed of the α subunit PTAR1 and the β subunit RabGGTB, whose β subunit is the same as that of GGTase-II.^21^ FBXL2 was the first identified substrate to undergo dual geranylgeranylation catalysed by GGTase-III, although it can also be regulated by GGTase-I to undergo monogeranylgeranylation.^253^ A crystal structure analysis revealed an extensive multivalent interface specifically shaped between FBXL2 and PTAR1, demonstrating that the recognition of FBXL2 by GGTase-III was mediated by PTAR1 and substrate–enzyme specificity.^253^ In addition, the Golgi SNARE protein Ykt6 is another substrate for GGTase-III, which is responsible for shifting a geranylgeranyl group to mono-farnesylated Ykt6 to generate geranylgeranyl-farnesyl Ykt6 for proper function.^254^ Mono-farnesylated Ykt6 prevents the Golgi SNARE complex from assembling properly, thus compromising Golgi structure and function.^254^

**Fig. 7:**
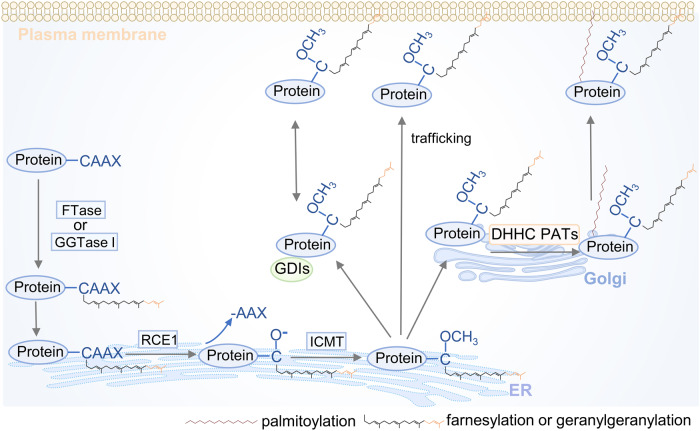
The catalytic process of protein S-prenylation and postprenylation reactions. Most substrate proteins have a characteristic CAAX motif, which is the enzyme recognition site. First, a 15-carbon farnesyl or a 20-carbon geranylgeranyl isoprenoid lipid is attached to the cysteine residues on the CAAX motif of the substrate by FTase or GGTase-I, respectively. Then, the prenylated proteins translocate to the ER and undergo removal of the AAX motif by RCE1 and methylation by ICMT. After that, proteins such as Ras may be directly trafficked to the plasma membrane or undergo additional S-palmitoylation. Other proteins, such as RHO GTPases, bind to RHO guanine nucleotide dissociation inhibitors (GDIs) to assist in their trafficking. GDIs guanine nucleotide dissociation inhibitors, ER endoplasmic reticulum

Before trafficking to the PM, most CAAX proteins undergo three stages: initial prenylation, endoproteolysis, and final methylation (Fig. 7). Some proteins may also undergo additional S-palmitoylation (see the S-palmitoylation section). Endoproteolysis refers to the removal of AAX amino acids from proteins on the surface of the ER after the attachment of isoprene to its C-terminal cystine residue, and the enzyme responsible for this process is RCE1, which was initially discovered in yeast.^255^ Regarding the proteolytic mechanism, earlier studies have suggested that RCE1 belongs to the cysteine protease family, and some have hypothesised that RCE1 is a membrane-bound metalloproteinase, but none of these theories are able to adequately explain its hydrolysis mechanism.^256,257^ The crystal structure of RCE1 orthologues from *Methanococcus maripaludis* (MmRce1) shows the involvement of glutamate-activated water and an oxyanion hole,^258^ which provides a glimpse into the mechanism of protein hydrolysis in *Homo sapiens* RCE1. However, crystal structural data on eukaryotic enzymes are also urgent to elucidate their mechanisms since the primary structure and topology of RCE1 differ among species.^258^ Methylation refers to the ICMT-mediated methylation of the exposed carboxyl terminus of farnesylated proteins after the removal of AAX amino acids, a process that increases protein membrane affinity by neutralising the negative charge of prenylcysteine.^22,259^ Similar to RCE1, ICMT was first discovered in yeast as STE14, and ICMT is restricted to the ER.^22,260^ Topology and mutational analysis of human ICMT revealed that some of the conserved amino acid clusters located on transmembrane fragments altered enzyme activity upon mutation, suggesting that they may be involved in substrate binding and/or catalysis.^259,261^ In the whole process of protein prenylation, it has been hypothesised that the final ICMT-catalysed methylation is reversible. Much of the speculation links to the discovery that unmethylated CAAX proteins have been observed in different cells.^262–264^ Furthermore, the carboxylesterase CES1 was identified in MDA-MB-231 cells, and knockdown of CES1 significantly reduced the amount of unmethylated RhoA protein, suggesting that CES1 is a prenylcysteine-specific methylesterase.^265^ However, the methylation status of other Rho family members was not affected by CES1, implying that either CES1 is highly selective for substrates or that only a fraction of prenylated proteins can achieve reversible methylation, which is a question worthy of further investigation.

### Physiological function

#### Intracellular trafficking and membrane localisation

For proteins exemplified by the Ras protein, accurate localisation on the PM is crucial. Cytosolic proteins, which start as soluble entities lacking hydrophobic sequences, rely on prenylation to effectively associate with the membrane. The Ras proteins have extensively showcased this property. Once prenylated, proteins such as N-Ras, H-Ras, and K-Ras4a undergo palmitoylation, they are anchored to the PM via two hydrophobic moieties. K-Ras4b, on the other hand, anchors to the PM using its farnesyl group combined with a lysine-rich polybasic sequence.^266^ Both nonfarnesylated mutant Ras and blocked isoprenoid biosynthesis can impair protein PM anchoring.^96,242^ In addition to targeting the PM, directing the positioning of proteins to different compartments is also one of the effects of prenylation. For instance, upon viral infection, Rac1 of the Rho family undergoes geranylgeranylation and subsequent palmitoylation and is subsequently transported to the mitochondria-associated endoplasmic reticulum membrane (MAM), where Rac1 continues to limit the interaction of mitochondrial antiviral signalling proteins with the E3 ligase Trim31, thereby inhibiting MAVS ubiquitination, aggregation and activation to prime the antiviral immune response.^267^ In addition, NGF-induced geranylgeranylated Rac1, along with other prenylated proteins in sympathetic axons, is recruited to TrkA-harbouring endosomes to assist receptor trafficking, which is critical for axonal growth during neuronal development.^268^ Previous studies have also assessed the importance of the three processing steps (prenylation, protein hydrolysis and methylation) of CAAX proteins for targeting the PM. Studies of K-Ras4b from rabbit reticulocytes revealed that compared with farnesylation alone, a twofold increase in membrane binding from the protein hydrolysis step and a further twofold increase in membrane binding from methylation.^269^ In addition, it was also found that prenylation alone was not sufficient for rab4 and Rab5 to achieve membrane association compared to their maturation forms which underwent the full three modification stages.^270^ For these proteins, hydrolysis-induced conformational changes that modify membrane binding and neutralise the negatively charged ionised carboxyl group induced by methylation are important factors responsible for increasing membrane affinity.

#### Protein–protein interactions

While the primary focus of many studies has been on the anchoring of proteins to membranes, protein prenylation plays a pivotal role in mediating protein–protein interactions as well. A classic example is that prenylation affects the binding of K-ras and SOS. SOS forms a complex with prenylated K-Ras4b and catalyses its guanine nucleotide exchange, whereas non-pernylated K-Ras4b is not able to form a complex with SOS.^271^ Furthermore, replacing the farnesyl with a geranylgeranyl moiety does not influence the binding between SOS and K-Ras. This observation indicates that the length of the attached lipid is not a limiting factor in their interaction.^271^ Among the various proteins that can interact with Ras, PDEδ is also one of the most extensively studied. The farnesylated Ras protein on the PM is bound and solubilized by PDEδ through its GDI-like pocket and unloaded at the perinuclear membrane with the assistance of Arl2. It is then captured in the recycling endosome (RE), from which it recovers its localisation on the PM via vesicular transport, which results in a PDEδ-regulated dynamic membrane distribution of Ras, therefore enhancing its signalling capacity.^272,273^ The crystal structure of the human K-Ras4b-PDEδ complex reveals the details of the interaction, and a 5-amino-acid-long sequence motif (Lys–Ser–Lys–Thr–Lys) in K-Ras4b was found to be particularly important for the interaction.^274^ In certain scenarios, nonprenylated proteins might exhibit a higher affinity for interactions than their prenylated counterparts. One example of this is Rac1. Nonprenylated Rac1 actively interacts with its effectors Iqgap1 and Tiam1 to support GTP loading and cytokine production, while the prenylation of Rac1 limits these interactions.^275^ Thus, promoting Rac1 prenylation could restrain pro-inflammatory signalling and innate immunity by hindering the interactions of Rac1 with Iqgap1 and Tiam1.

#### Stability and protein degradation

Prenylation also plays a pivotal role in modulating protein stability. For example, farnesylation enhanced the stability of Ykt6, as a faster degradation rate was observed in nonfarnesylated Ykt6 than in farnesylated Ykt6, and this increased stability is closely related to the more compact and stable structure induced by farnesylation.^276^ In certain instances, the influence of prenylation on protein stability is more indirect. For instance, geranylgeranyation of the Rho protein CDC42 is crucial for the interaction with its chaperone protein RHOGDI.^277^ The binding of RHOGDI to Rho protein not only facilitates correct membrane association of the Rho protein but also protects it from degradation.^278^ In the case of CAAX proteins, methylation of prenylated cysteine groups at their C-terminus significantly impacts their stability. Loss of methylation exerts distinct effects on different methyl-accepting proteins. An experiment conducted in the RAW264.7 macrophage line showed that both the RhoA and CDC42 half-lives were shortened to varying degrees when methylation was inhibited.^279^ Another experiment in fibroblasts showed that when ICMT was inactivated, the levels of total RhoA and GTP-bound RhoA protein were only 5–10% of those of controls.^280^ However, another experiment performed in human TM cells appears to be contradictory to the previous observation, as promoting RhoA geranylgeranylation by the addition of geranylgeranyl pyrophosphate (GGPP) decreases its half-life and promotes its degradation through the 20 S proteasome pathway.^281^ Given that this study focused on initial geranylgeranylation and did not explore the role of methylation, experiments involving prenylated cysteine methylation changes should be included to determine whether methylation affects RhoA degradation. Notably, the lack of ICMT indeed increased the K-Ras half-life, demonstrating that the absence of methylation contributes to K-Ras stability.^280^ More investigations are needed to explore the effect of the three catalytic steps of CAAX protein maturation on protein stability.

#### Signal transduction

**Fig. 8:**
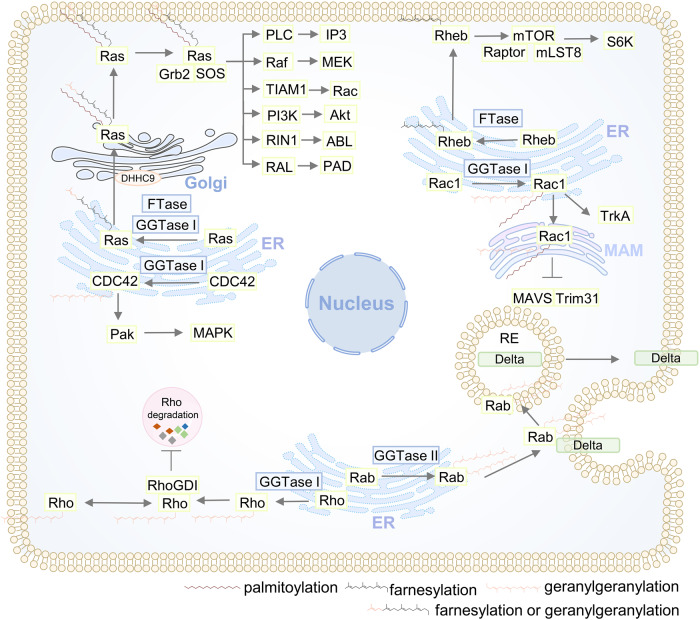
S-prenylation is involved in the regulation of multiple cellular signal transduction pathways. We briefly summarised several well-established signalling pathways of the main small GTPase family. The prenylation and post prenylation of these proteins often occur on the ER. K-Ras and N-Ras can be either farnesylated or geranylgeranylated, while H-Ras can only be farnesylated. N-Ras, H-Ras and K-Ras4a undergo S-palmitoylation before anchoring at the plasma membrane and activating downstream signalling. Other proteins, such as Rho, can switch membrane-bound states through binding to RhoGDI. Overall, protein prenylation mediates downstream signalling by regulating protein trafficking and protein‒protein interactions. ER endoplasmic reticulum, MAM mitochondria-associated endoplasmic reticulum membrane, RE recycling endosome

Given the widespread occurrence of prenylation among cellular signalling proteins, a multitude of signal transduction pathways are regulated by protein prenylation (Fig. 8). For instance, after being prenylated, Ras properly localises on the PM and binds to the receptor protein Grb2 to form a complex, which subsequently recruits SOS to cause the conformation change of Ras, allowing it to dissociate from GDP and binding to GTP and be activated. Subsequently, activated Ras activates various effectors, such as PI3K, Raf, and PLC, resulting in the activation of distinct signalling cascades.^282^ Maintaining a balance between farnesylation and geranylgeranylation is pivotal for optimal cellular function. Any imbalance can induce abnormal signal transduction, subsequently leading to pathological cellular alterations. For example, GGPPS is a key enzyme in the mevalonate pathway catalysing the synthesis of GGPP from FPP.^283,284^ Knocking down or pharmacologically inhibiting GGPPS results in reduced GGPP synthesis, and excess farnesyl pyrophosphate (FPP) can enhance the farnesylation of Rheb, which in turn overactivates mTORC1 signalling, thus leading to cardiomyocyte hypertrophy.^285^ In addition, the selective shaping of downstream pathways by farnesylation and geranylgeranylation is reflected in the regulation of the immune system. Fntb-mediated protein farnesylation regulates eTreg cell maintenance through activation of mTORC1 and ICOS signalling, while Pggt1b-mediated protein geranylgeranylation regulates eTreg cell differentiation through activation of Rac signalling.^286^ In addition to the regulation of Treg cells, farnesylation and geranylgeranylation also play their respective roles in regulating peripheral T cells. It has been found that Fntb-mediated protein farnesylation is indispensable for peripheral T-cell homeostasis, as peripheral T cells lacking Fntb exhibited a less activated phenotype and were susceptible to apoptosis. Pggt1b-mediated protein geranylgeranylation is responsible for the egress and trafficking of thymocytes, as Pggt1b deficiency blocks S1P1 signalling and Cdc42-Pak signalling, which are essential for thymocyte egress and trafficking.^287^ For the Rab protein, its dual geranylgeranylation, facilitated by GGTase-II, is vital for the proper trafficking and positioning of Notch signalling elements, such as Delta. Mistrafficking of Notch signalling components caused by the mislocalization of Rabs leads to Notch signalling defects.^288^

### Pathological implication

#### Cancer

The involvement of small GTPases, particularly Ras GTPases, in the carcinogenesis of diverse cancers is well-documented. Approximately 20% of human cancers harbour Ras subfamily mutations, which maintain Ras in a constitutively active GTP-binding conformation and subsequently overactivate downstream signalling to mediate tumorigenesis.^266^ In addition, other small G proteins, such as Rho, Rab and Arf subfamilies, are overexpressed in a variety of cancers and promote tumorigenesis by influencing intracellular regulatory processes.^266^ These molecules need to undergo prenylation to ensure their proper subcellular localisation to the PM and/or endomembranes before performing their oncogenic functions. For example, H-Ras can be farnesylated.^289^ K-Ras, N-Ras and RhoB can be either farnesylated or geranylgeranylated.^290^ RhoA, RhoC and CDC42 can be geranylgeranylated.^247^ RAB1 and RAB3b can be double geranylgeranylated.^291^ Disrupting the prenylation process can significantly hinder the oncogenic role of Ras. For example, inhibiting FTase activity with a selective inhibitor of FTase tipifarnib to suppress H-Ras prenylation showed that the proliferation, survival, and spheroid formation of H-Ras-mutant cells were impaired, and tumour stasis or regression was observed in head and neck squamous cell carcinoma H-Ras-mutant xenografts after treatment with tipifarnib.^292^ Similarly, treatment with tipifarnib in H-Ras-mutant fusion-negative rhabdomyosarcoma xenografts could reduce tumour growth by reducing H-Ras PM localisation and downstream signalling transduction.^289^ Overall, the discovery of the critical role of prenylation in regulating Ras function sheds light on the intricate mechanisms and regulatory networks in cancer development driven by Ras and provides a potential strategy targeting key links in protein prenylation.

#### Neurological disorders

The pathophysiology of numerous neurological disorders has been associated with protein prenylation. For example, elevated levels of FPP and GGPP were observed in the brains of AD patients, and suppression of FTase or GGTase-I could mitigate Aβ-associated neuropathology in an AD mouse model.^293^ In another study, upregulated expression of FT and H-Ras farnesylation was observed in AD brains and the upregulation of FT and H-Ras farnesylation was associated with mild cognitive impairment, indicating that abnormal regulation of protein prenylation is involved in the AD pathogenic cascade. Targeting protein prenylation by deleting FT to inhibit amyloid generation and overactivation of mTORC1 signalling could effectively alleviate memory impairment in AD model mice.^294^ In addition to AD, other neurological disorders, including Hutchinson-Gilford progeria syndrome (HGPS),^295^ multiple sclerosis,^296^ Niemann-Pick disease type C disease^293^ and Parkinson’s disease^297^ are subject to aberrant regulation of protein prenylation. As a rare genetic disease, the pathogenesis of HGPS is closely linked to point mutations in LMNA, which encodes lamin A.^298^ The mutant lamin A lacks its Zmpste24 site, preventing it from undergoing endoproteolysis. Instead, it remains consistently farnesylated and carboxymethylated, which leads to limited remodelling of the nuclear scaffold and increased blebbing, thereby accelerating premature ageing.^295^ Given that aberrant protein prenylation is involved in the pathology of many neurological disorders, in-depth investigations of the regulatory mechanisms of prenylation will provide new avenues for the development of effective therapeutic approaches for these neurological diseases.

#### Infectious diseases

A growing body of evidence underscores the important role of protein prenylation in the infection processes of multiple pathogens. As one of the pathogens causing pneumonia, *Legionella pneumophila* enters cells by phagocytosis and subsequently secretes effector proteins, which are prenylated by host FTase, anchor into the PM and escape from lysosomal fusion for sustained infection.^299^ Beyond bacteria, prenylation has been found in various viruses, including type 1 adenovirus, hepatitis D virus (HDV), influenza virus, and HIV, among others.^300^ In the hepatitis D virus, the delta virus large antigen is prenylated, which is essential to anchor to HBsAg and be packaged into virus particles for HDV particle formation.^301^ HDV replication can be attenuated by inhibiting geranylgeranylation.^302,303^ Furthermore, protein prenylation is also involved in the regulation of the survival and activity of several parasites, such as *Plasmodium falciparum*.^304^ Researchers found that *Plasmodium falciparum* survival during heat or cold shock is dependent on the regulation of protein prenylation, which governs the membrane binding of HSP40 to the ER and interaction with its client proteins, thereby controlling thermotolerance. Inhibition of protein prenylation would result in *Plasmodium falciparum* being sensitive to otherwise nonlethal temperature stresses.^304^ Taken together, these research findings highlight the crucial role of protein prenylation regulation in ensuring the viability of an extensive array of pathogens, including bacteria, viruses, and parasites.

#### Other diseases

A variety of diseases affecting several systems have been linked to abnormalities in protein prenylation, such as diabetes, obesity, fatty liver disease, male infertility, chronic heart failure, and inflammatory bowel disease.^305–307^ Aberrant expression of GGPPS was observed in diabetes, fatty liver disease, male infertility and cardiac hypertrophy, which disrupted the balance of protein farnesylation and geranylgeranylation. For instance, highly expressed GGPPS in adipocytes enhanced K-Ras geranylgeranylation to activate Ras/MAPK/Erk1/2 signalling, which inhibited insulin signalling and led to insulin resistance.^308^ However, downregulated GGPPS in the heart increases Rheb farnesylation to activate mTORC1 signalling, which further leads to cardiac hypertrophy and heart failure.^285^ By adjusting the levels of GGPP and FPP, GGPPS can play a pivotal role in governing both physiological and pathological cellular processes. Therefore, targeting the aberrant expression of GGPPS in diseases is a promising therapeutic direction for the direct inhibition of protein prenylation.

### Therapeutic targets and clinical research progress

#### Targeting the mevalonate pathway

Given that the lipid substrates needed for protein prenylation, FPP or GGPP, are synthesised through the mevalonate pathway, inhibition of key catalase enzymes in the mevalonate pathway to limit the synthesis of FPP or GGPP is expected to be a therapeutic target for diseases (Tables 1 and 2). Statins are renowned agents that selectively inhibit HMG-CoA reductase, consequently diminishing the biosynthesis of downstream molecules such as FPP and GGPP.^283^ Although statins were initially used to control plasma cholesterol levels and prevent cardiovascular disease, studies have revealed that statins also potentially exert a therapeutic effect on cancer, neurological disorders and other diseases.^309,310^ Regarding antitumour effects, considerable evidence suggests that statins can inhibit tumour proliferation and metastasis by disrupting farnesylation and geranylgeranylation of small GTPases.^311,312^ For instance, statin-mediated inhibition of K-Ras prenylation induces immunogenic cell death by enhancing ER stress, thereby stimulating a strong CD8 T-cell immune response against K-Ras mutant tumours.^313^ However, statins mediate antitumour effects not only by disrupting protein prenylation but also by lowering cholesterol levels, modulating autophagy and inducing ferroptosis and pyroptosis to promote the apoptosis of tumour cells and inhibit their proliferation and invasion.^314–317^ Despite preclinical and epidemiological data indicating that statins may have antitumour properties, clinical trial results show that statins exert varying therapeutic effects on different malignancies. For instance, a prospective cohort study of breast cancer patients found that lipophilic statin use was associated with a reduced risk of recurrence in breast cancer patients.^318,319^ However, a randomised adjuvant chemotherapy clinical trial in stage III colon cancer patients showed that compared to nonusers, patients who received statin treatment during and after adjuvant chemotherapy had similar survival outcomes. The survival outcomes were similar among users regardless of K-Ras mutation.^320^ Similarly, other clinical trials in advanced gastric cancer patients found that the addition of simvastatin or pravastatin to chemotherapy does not improve survival outcomes.^321,322^ In conclusion, careful consideration of tumour specificity and indications, as well as multiple pharmacological properties, is needed before applying statins in the clinical treatment of cancer. Another class of drugs that target FPP synthase within the mevalonate pathway includes bisphosphonates, with zoledronic acid being a notable example. It is widely used to treat benign and malignant bone diseases by disrupting protein prenylation.^323^ In addition to bone disease, bisphosphonates have been explored for the treatment of cancer, Hutchins-Gilford progeria, AD and cerebral cavernous malformations, and to some extent, such therapeutic potential is dependent on inhibiting protein prenylation, although it also induces apoptosis and exerts antiangiogenic and antimigratory effects.^324,325^ One noteworthy aspect for bisphosphonates is that bisphosphonates prefer to inhibit protein geranylgeranylation rather than protein farnesylation, although the synthesis of both GGPP and FPP is reduced,^326,327^ suggesting that bisphosphonates may have greater efficacy in diseases triggered by protein geranylgeranylation. Clinical evaluation of bisphosphonates mainly focuses on bone diseases or cancers with a high tendency to develop bone metastasis, and some clinical trials have also been conducted to test bisphosphonates for the treatment of progeria. The therapeutic applications of bisphosphonates in other diseases are equally worthy of exploration.

#### Targeting protein prenylation

Methods for targeting prenylation include inhibiting FTase for protein farnesylation or GGTase for protein geranylgeranylation. The former is generally achieved by FTase inhibitors (FTIs), which are broadly classified into four categories. The first category is FPP analogues that bind with FTase.^328^ The second category of FTIs includes BMS-184467 and BMS-185878, which are able to bind the structural motifs of farnesyl pyrophosphate and CAAX tetrapeptide.^329^ The third category is peptidomimetic compounds that mimic the CAAX box, such as GGTI-2418.^330^ The last category is nonpeptidomimetic enzyme-specific inhibitors, which are identified from high-throughput screening efforts such as tipifarnib, lonafarnib and BMS-214662.^331,332^ While FTIs showed promising antitumour effects in preclinical studies, their performance in clinical trials was less than satisfactory.^333^ Most clinical trials have shown that monotherapy with FTIs has no or little efficacy in the treatment of haematopoietic cancers or solid tumours.^333^ One of the reasons for the discrepancy between laboratory findings and clinical trial results is that in tumours with predominantly K-Ras and N-Ras mutations, K-Ras and N-Ras can escape FTI-mediated inhibition through alternative geranylgeranylation and thus continue to retain signalling activity.^334^ This finding suggests that tumours driven by H-Ras mutations might be more susceptible to FTIs, given that H-Ras undergoes only farnesylation. To support this hypothesis, a phase II clinical trial in patients with recurrent and/or metastatic head and neck squamous cell carcinoma (HNSCC) showed that HNSCC patients with H-Ras mutations had a 55% objective response after receiving tipifarnib.^335^ Another phase II clinical trial in advanced refractory uroepithelial cancer patients with H-Ras mutations also found that response was observed in 5 out of the 12 evaluated patients after treatment with tipifarnib.^336^ For other tumours, FTIs in combination with other treatments can improve treatment efficacy to some degree. For example, the combination of the FTI tipifarnib with gemcitabine and cisplatin was observed to be well-tolerated and showed antitumour activity in a phase I clinical trial in patients with advanced solid tumours.^337^ However, the side effects of FTI application should not be ignored. Common adverse events include gastrointestinal toxicity, myelosuppression, fever, hypokalaemia, hyperbilirubinemia, peripheral neuropathy, skin rash and others,^338–341^ and the majority of adverse events are associated with the FTI dose. Even though clinical trials for cancer treatment were less promising, the application of FTIs showed encouraging results in treating HDV infections, as well as progeria and progeroid laminopathies. In 2020, lonafarnib was approved by the FDA for the treatment of HGPS and processing-deficient progeroid laminopathies, and clinical development of lonafarnib for the treatment of HDV infection is also underway.^342^ As protein geranylgeranylation is catalysed by GGTases, the development of GGTase inhibitors (GGTIs) represents a promising strategy for diseases with protein geranylgeranylation. For instance, a genetic study demonstrated that inactivation of GGTase-I activity could reduce lung tumour formation and improve the survival of mice with K-Ras-induced cancer.^343^ Combined treatment with FTI and GGTI produces a synergistic cytotoxic effect, further promoting an increase in cancer cell apoptosis, which is expected to improve treatment efficacy in FTI-resistant cancers.^344,345^ Although multiple GGTIs have shown antitumour activity in preclinical studies, only the maximum tolerated dose of GGTI-2418 was assessed in patients with advanced solid tumours.^330,346^ Beyond cancer treatment, GGTIs have potential applications in addressing certain infectious and inflammatory diseases.^267,347^ In addition to the combination of FTIs and GGTIs, agents inhibiting both FTase and GGTase-I, such as FGTI-2734 and L-778,123, have been developed. The dual inhibitor FGTI-2734 impedes tumour cell growth and is expected to overcome the barrier of K-ras resistance.^348^ In addition, FGTI-2734 inhibits mast cell-dependent allergic reactions, which is a promising therapeutic strategy for allergic diseases.^349^ L-778,123 has been assessed in preclinical and clinical studies. The results showed that L-778,123 has a radiosensitizing effect in cell lines with Ras activation and produces a clinical response in patients with advanced solid tumours.^350,351^

#### Targeting protein post prenylation

Researchers have been increasingly interested in targeting postprenylation processes such as endoproteolysis and methylation, given that complete catalysis is vital for the functioning of most CAAX proteins. When RCE1 is conditionally deleted, Ras loses its capability to participate in endoproteolysis and methylation, leading to the partial mislocalization of K-Ras and H-Ras, which retards cell growth, reduces Ras-induced transformation, and sensitises tumour cells to farnesyltransferase inhibitors.^352^ Inactivating ICMT activity has stronger implications in disrupting oncogenesis than activating RCE1. Inactivation of ICMT inhibits not only cell growth and K-Ras-induced oncogenic transformation but also the transformation of the oncogenic form B-Raf.^280^ In addition, ICMT inhibition by the compound UCM-1336 suppresses the activity of the four Ras isoforms, which further suggests that ICMT is a valuable target.^353^ Tumours induced by Ras and Rho GTPases are targets for RCE1 and ICMT inhibitors,^247^ but the efficacy of such inhibitors in Rheb-induced tumours is likely to be limited, as RCE1 and ICMT inhibitors do not block Rheb function.^354^ Although several small-molecule inhibitors targeting RCE1 have been identified, with some causing Ras mislocalization, studies on their therapeutic applications remain limited.^258^ A variety of ICMT inhibitors have been developed targeting Ras-related cancers, positioning them as potential anticancer drugs.^355^ Cysmethynil, a representative ICMT inhibitor, has demonstrated antitumour activity in a wide range of tumours. For example, the treatment of colon cancer cells with cysmethynil resulted in Ras mislocalization and impaired epidermal growth factor signalling, thereby inhibiting cell growth.^356^ Treatment of prostate cancer cells with cysmethynil leads to the accumulation of cells in the G (1) phase and cell death and hinders tumour growth.^357^ Subsequent development of cysmethynil derivatives such as analogue 15 and Compound 8.12 further improved its solubility and PAMPA permeability, increasing the potency of the compounds and making them more suitable for preclinical and clinical development.^358,359^ Beyond cancer treatment, ICMT knockout enhances the survival rates of HGPS model mice. Furthermore, using the ICMT inhibitor C75 has been found to delay ageing and stimulate the proliferation of HGPS cells and Zmpste24-deficient mouse fibroblasts, suggesting that ICMT inhibitors also have therapeutic potential in the treatment of HGPS.^360^ Although ICMT inhibitors are not yet in clinical studies, their therapeutic potential should not be underestimated.

### Glycosylphosphatidylinositol (GPI) anchor

#### Biosynthesis and maturation of GPI-anchored proteins (GPI-APs)

**Fig. 9:**
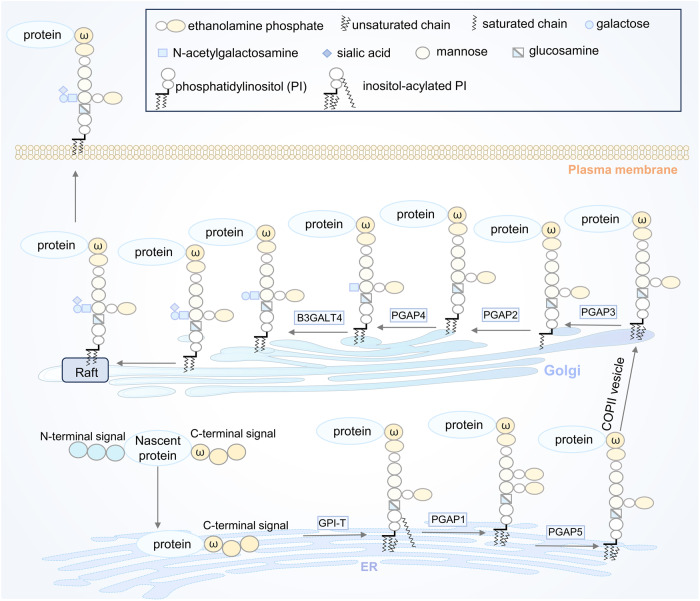
Biosynthesis and maturation of GPI-anchored proteins (GPI-APs). N Nascent proteins first translocate to the ER following the N-terminal signal. After arriving at the ER, the N-terminal signal is removed. Subsequently, GPI-T recognises the amino acid at the ω position and catalyses the attachment of GPI at the ω position, followed by fatty acid remodelling of GPI with or without GalNAc side chain modification of the ER and Golgi. Finally, mature GPI-APs are trafficked to the plasma membrane and associate with raft microdomains. ER endoplasmic reticulum

GPI is primarily synthesised in the ER through a series of 11 consecutive reactions.^23^ The biosynthesis and maturation of GPI-APs is also a sophisticated process that mainly involves the major steps of precursor protein localisation to the ER, GPI attachment, and GPI maturation (Fig. 9). All GPI-anchored proteins possess a signal sequence, with ~20 hydrophobic amino acids at the N-terminus, that facilitates translocation across the ER membrane.^361,362^ After localisation of the precursor protein to the ER, GPI attaches to the C-terminus of the precursor protein, and the ω site refers to the amino acid residue to which GPI attaches, usually Ser, Asn, Asp, Ala, Gly, Cys and Thr.^363,364^ The signal peptide that mediates C-terminal GPI attachment is a nonconserved sequence of 20–30 amino acids starting from the ω + 1 amino acid, which includes a segment of ~10 hydrophilic amino acids and a segment of ~20 hydrophobic amino acids.^361^

The GPI-transamidase (GPI-T) complex, residing in the ER and comprising five subunits (PIGK, GPAA1, PIGT, PIGS and PIG-U), attaches the GPI anchor to proteins.^365^ PIGK is considered to be the catalytic subunit.^366,367^ Structural analysis of the GPI-T complex suggests that PIGK exhibits caspase-like folding and forms an extensive interaction with the other subunits, GPAA1, PIGT and PIGS, and an open cavity located directly beneath the PIGK subunit accommodates a lipid moiety of the GPI molecule and supports its binding to the GPI substrate.^368^ Regarding the biosynthesis process of nascent GPI-AP, the GPI-T complex initially cleaves the peptide bond between ω and ω + 1 amino acid of the precursor protein via its PIGK subunit, removing the GPI attachment signal peptide, and then connects the ω amino acids via a thioester bond to form a substrate–enzyme intermediate. Subsequently, the amino group of the terminal EtN of GPI attacks the thioester bond and completes the transfer of GPI by transamidation.^23,369^ A recently identified subsite featuring human GPI-T structures further revealed its broad proprotein specificity and activation mechanis.^370^ The selectivity of GPI-T mainly depends on the ω-site, which is restricted by a deep pocket consisting of various residues to define ω-residue preferences. The autoinhibitory loop of PIGK regulates GPI-T activation through a drastic conformational rearrangement. Prior to maturation, GPI molecules of nascent GPI-APs need to undergo remodelling reactions during transport from the ER to the PM. Generally, in the ER, the acyl chain attached to the inositol ring of GPI is removed by PGAP1,^371^ and the EtNP attached to the second mannose is removed by PGAP5.^372^ Following this, GPI-APs are recruited to COPII-coated transport vesicles at the ER exit in the presence of p24 family proteins and then transported to the Golgi.^373^ Upon arrival at the Golgi, the PGI molecule undergoes fatty acid remodelling, which is characterised by the removal of the unsaturated 2-acyl chain by PGAP3, followed by the transfer of a saturated chain back to the sn2-position and acylation by PGAP2.^374,375^ The two long saturated lipid chains formed by fatty acid remodelling of GPI-APs contribute to trans-bilayer interactions with long acyl-chain-containing phosphatidylserine and are essential for GPI-APs incorporation into lipid rafts.^374,376^ After FA remodelling, some GPI-APs also undergo GalNAc side chain modifications on the Golgi before trafficking to the PM and associating with lipid rafts. For example, βGalNAc is transferred to the 4 position of Man1 as a side chain and is further extended by β1-3Gal and Sia.^23^ The physiological roles of such side chain modifications remain largely unknown and need to be further elucidated.

### Physiological function

#### Protein trafficking and lipid raft association

The attachment of GPI anchors gives GPI-APs unique trafficking characteristics and offers a solid PM anchor, setting the stage for GPI-APs to play various biological roles. GPI-APs undergo numerous regulatory changes and modifications throughout the ER-Golgi-PM transport process. If any of the key modifications are abnormal, their transport and lipid raft associations are disrupted. For example, when PGAP3 is deficient, unsaturated FAs remain on PGI-APs, preventing GPI-APs from associating with membrane microdomains and thereby reducing PGI-AP levels on the cell surface.^374^ Similarly, defects in PGAP3 decrease stable surface expression of a variety of GPI-APs.^377^ For proteins that naturally have both GPI-anchored and transmembrane isoforms, there is no functional difference between them.^378^ However, for other GPI-APs, GPI anchoring is an important determinant of GPI-AP lipid raft association, and when replacing GPI with the transmembrane region from a nonraft protein, the majority of GPI-APs lose their raft association ability.^379,380^

#### Signal transduction

**Fig. 10:**
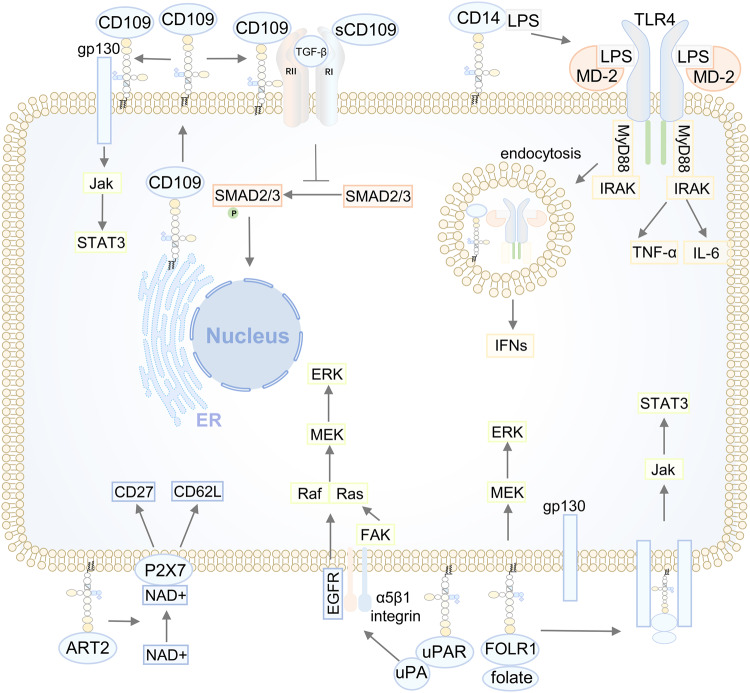
Glycosylphosphatidylinositol anchor proteins (GPI-APs) play a role in several cellular signal transduction pathways. In this section, we describe several classical GPI-APs and their roles in cellular signal transduction. CD109 negatively regulates TGF‐β signalling, while it can activate the Jak-STAT3 pathway to drive tumour metastasis. CD14 can activate TLR4 to trigger signalling pathways, including INF, tnf-α and IL-6 signalling, to initiate the immune response. Folate binds to FOLR1 to activate MEK/ERK signalling and the Jak-STAT3 pathway. ART could trigger NAD + -induced cell death. uPAR binds to Upa to stimulate the association of EGFR and β1 integrin to activate MEK/ERK signalling. ER endoplasmic reticulum

In mammalian cells, in addition to acting as membrane anchors, GPI-APs are capable of transmitting a variety of signals and regulating multiple intracellular and extracellular responses such as immune responses, cell proliferation and metastasis (Fig. 10). One of the most typical representatives of mediated signal transduction is CD molecules. For instance, CD109 is a GPI glycoprotein that is widely expressed on immune cells, myoepithelial cells, and basal cells in normal tissues as well as various tumour cell lines.^381–384^ CD109 can be expressed on the cell surface and enriched in lipid rafts or extracellularly released with exosomes.^385^ It can also be a secreted protein presented as a truncated form harbouring the 180-kDa CD109 subunit.^386^ It was shown that both GPI-anchored CD109 and soluble CD109 could bind to the TGF-β receptor, thus inhibiting TGF-β-induced SMAD2/3 phosphorylation and further hindering its regulatory function in the cell nucleus.^387^ In addition to negatively regulating TGF‐β signalling, CD109 is involved in EGFR-Akt-mTOR signalling, JAK-STAT3 signalling and YAP/TAZ signalling to mediate tumour proliferation and metastasis.^388–390^ Another typical GPI-anchored glycoprotein, CD14, which is widely expressed on the cell membrane surface of myeloid lineage cells, is the first pattern recognition receptor (PRR) identified to bind directly to LPS and is an important regulator of innate immunity.^391,392^ After recognising and binding LPS, CD14 transfers LPS to MD-2 of the TLR4/MD-2 complex to promote TLR4 dimerisation. TLR4 dimerisation further induces the formation of Myddosomes with MyD88, which triggers a signalling cascade leading to activation of the NF-κB and MAPK pathways.^392,393^

#### Other physiological functions

Given the diversity of GPI-anchored proteins, which involve enzymes, adhesion molecules, receptors, antigens and others, each GPI protein performs its respective physiological functions. For instance, ADP-ribosyltransferase ART2 is a GPI-anchored exonuclease that uses free NAD+ to mono-ADP-ribosylate the P2X7 receptor on CD8 T cells, leading to NAD-induced cell death and a reduction in the P2X7R + CD8 + T-cell subpopulation in the TME.^394,395^ NCAM is a typical adhesion molecule that regulates cell–cell adhesion by homophilic and heterophilic interactions and is involved in the regulation of the development and plasticity of the nervous system.^396^ The receptors expressed on the cell membrane surface, in addition to mediating signal transduction, are also involved in nutrient uptake. Folate is one of the essential vitamins for cell growth, and its uptake is mediated through GPI-anchored folate receptors (FOLR1). When the GPI anchor of the folate receptors was replaced with transmembrane and cytosolic portions, the uptake efficiency of folate was significantly reduced, indicating that PGI-anchored protein-mediated nutrient uptake is indispensable and plays an important role in modulating JAK-STAT3 signalling and ERK1/2 signalling.^397–399^

### Pathological implication

#### Cancer

Research indicates that GPI-anchored proteins are crucial in various cancers, influencing cancer cell adhesion, migration, angiogenesis, extracellular matrix (ECM) remodelling, and immune responses. GPAA1, a type of GPI-AP, is overexpressed in various cancers.^400–402^ Thorough exploration of its tumorigenic function and underlying mechanism in gastric cancer revealed that GPAA1 enhanced lipid raft formation to support the interaction between EGFR and ERBB2, followed by further activation of downstream Akt signalling to enhance the proliferation of cancer cells.^403^ Another GPI-AP, uPAR, is prevalent in numerous cancers, encompassing solid tumours, leukaemias, and lymphomas. One factor that drives tumour progression by uPAR is its ability to regulate ECM proteolysis. After binding to uPAR, uPA cleaves plasminogen to generate the active protease plasmin, which further activates MMPs to degrade ECM components and promote tumour cell migration and invasion.^404^ In addition, uPAR regulates intracellular signalling pathways to control cell proliferation. uPAR–α5β1 integrin interaction signals to FAK and then activates EGFR, and uPAR–β1 integrin–EGFR signalling enhances ERK activation to promote cancer cell proliferation.^405^ The importance of these GPI-APs in cancer also implies that GPI-T may act as an oncogene in tumour development. Abnormal expression of each GPI-T subunit has been observed in diverse cancer types to be involved in the regulation of GPI-AP expression on the cell surface,^365^ indicating the value of GPI-T as a potential biomarker and therapeutic target.

#### Haematological disorders

Paroxysmal nocturnal haemoglobinuria (PNH) is one of the most representative haematological disorders due to GPI-AP defects, with the main clinical manifestations of haemolytic anaemia, thrombosis and bone marrow failure in some cases. In PNH, the most common cause of GPI-AP defects is somatic mutations in the X-linked gene PIGA, as it is a critical gene encoding one of several enzymes that is needed for the biosynthesis of GPI anchors.^406^ Such mutations lead to defective surface expression of various GPI-anchored proteins, including CD55 and CD59, which function as complement regulators to prevent activated complement toxicity.^407^ In contrast to normal red blood cells, CD55-defective and CD59‑defective PNH red blood cells lack self-protective complement regulatory factors and thus are highly sensitive to complement activation, which results in intravascular haemolysis.^408^

#### Neurological disorders

GPI for GPI-APs, such as PrPC, plays a crucial role in protecting neurons, which is reflected in the fact that GPI is indispensable for PrPC to protect and repair the neuronal cytoskeleton and neuronal communication.^409^ Many neurological disorders exhibit pathological shifts stemming from downregulated cell surface GPI-AP expression, often linked to mutations in genes responsible for GPI-AP biosynthesis and remodelling. For instance, mutations in PGAP3 impair the GPI-anchor fatty acid-remodelling step, resulting in GPI-AP deficiency, which affects normal neural and embryonic development, leading to intellectual disabilities.^410^ Sequencing analysis of a family with encephalopathy and nonspecific autosomal-recessive forms of intellectual disability revealed the homozygous 3 bp deletion of another PGI-APs remodelling gene, PGAP1.^411^ In addition, biallelic splice mutations in ARV1 have been detected in patients with early infantile epileptic encephalopathy, as a reduction in ARV1 levels leads to impaired GPI-anchor synthesis, which hampers normal neurological development and leads to symptoms such as early-onset epilepsy.^412^ Furthermore, mutations in other genes regulating the biosynthesis of GPI-APs, such as GPI-T,^413^ PGAP2,^414^ PIGB,^415^ C18orf32^416^ and others, have been detected in a variety of patients with neurodevelopmental anomalies, indicating that the downregulated expression of cell surface GPI-APs due to mutations in genes regulating GPI-AP remodelling is a common phenomenon in neurological disorders.

#### Infectious diseases

Many of the GPI-APs in pathogenic protozoans and fungi act as virulence factors involved in survival and infection within distinct host environments. A prime example is the GPI-anchored variant surface glycoprotein (VSG) in *Trypanosoma brucei*, aiding in immune evasion.^417^ Other GPI-APs of *Trypanosoma brucei*, such as Procyclin, are implicated in protease resistance, while GPI-PLC and GP63 are implicated in VSG shedding. TfR is implicated in transferrin uptake.^418^ Similarly, various GPI-APs, such as MSP-1, MSP-2, MSP-4, MSP-5 and MSP-10, are spread over the surface of Plasmodium merozoites, contributing to parasite survival and invasion.^419^ In addition to protozoans, fungi also require GPI-APs to support biological processes such as cell wall construction,^420^ selective adhesion and invasion.^421,422^

### Therapeutic targets and clinical research progress

Due to the intricate processes from the initiation to maturation of GPI-APs, targeting their biosynthesis for therapy is both promising yet challenging (Tables 1 and 2). MSLN is a GPI-AP overexpressed in multiple tumour tissues and an attractive target for CAR T-cell therapy. Deficiencies in GPI-anchor biosynthesis by downregulating MSLN expression resulted in MSLN CAR T-cell resistance in pancreatic cancer.^423^ Among the many regulatory enzymes, the GPI-T subunit PIG-U was pinpointed as an oncogene for bladder cancer in 2004,^424^ which sparked the interest of researchers to explore whether other subunits also play oncogene roles and whether they have therapeutic value for cancer treatment. Moreover, most GPI-T subunits overexpressed in a wide range of tumours and have potential as anticancer targets.^425^ Unfortunately, the development of relevant inhibitors has not progressed remarkably. In addition to targeting the modification of GPI attachment to proteins, targeting GPI itself is also a therapeutic strategy. Although the GPI core structure is conserved among organisms, species-specific differences have been observed in the additional modifications to GPI structures and biosynthetic pathways, suggesting that it can be exploited for the development of anti-infectious drugs. GPI structures and GPI-APs play critical roles in the virulence of pathogenic protozoans and fungi. Research on hindering the protozoan GPI pathway has chiefly focused on GlcNAc-PI de-Nacetylase, which is essential for the second step of GPI biosynthesis. Salicylic hydroxamic acid (SHAM) has been identified as a nonsubstrate analogue inhibitor of the trypanosomal de-N-acetylase with high ligand efficiency.^426^ Regulatory enzymes Gwt1 and Mcd4 have been exploited for the development of antifungal inhibitors. Gwt1 is responsible for catalysing inositol acylation, while Mcd4 is responsible for catalysing phosphoethanolamine addition to first mannose. The identified Gwt1 inhibitors include BIQ, E1210 (APX001), Gepinacin, G365, G884 and Compound A1.^427^ Mcd4 inhibitors include M743 and M720.^428^ Among those inhibitors, APX001 which showed profound in vitro broad-spectrum antifungal activity, has entered phase I/II clinical trials and the results show that APX001 is safe, well-tolerated, and efficacious in participants with candidemia caused by *C. auris*.^429,430^ Species-specific differences in the pathogen GPI pathway create opportunities for the development of anti-infective drugs, and further optimisation of the potency, as well as the specificity of inhibitors, will be beneficial and expand their application.

## Cholesterylation

### Mechanism of cholesterylation

**Fig. 11:**
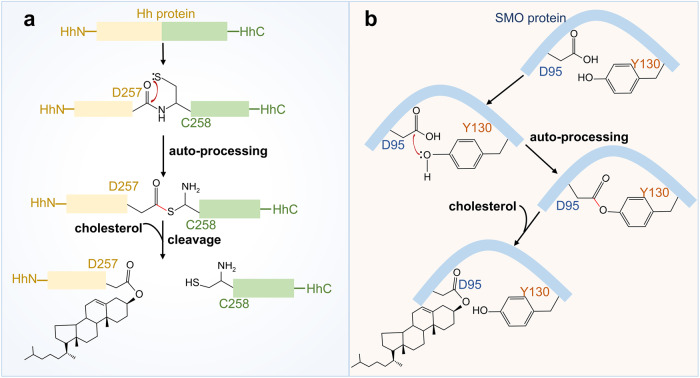
Mechanism of hedgehog (Hh) and smoothened (SMO) cholesterylation. **a** Autoprocessing nucleophilic attack between C258 of Hh and G257 induces the formation of a labile thioester intermediate. Subsequently, the activated intermediate undergoes cleavage, and cholesterol attaches to HhH to complete HhH cholesterylation. **b** The autoprocessing nucleophilic attack between D95 and Y130 of SMO induces the formation of a high-energy intermediate, followed by ester exchange from D95–Y130 to D95–cholesterol. C258: Cystine 258; G257: Glycine 257. D95: Aspartic acid 95; Y130: Tyrosine 130

Hedgehog (Hh) and smoothened (SMO) are proteins that undergo cholesterylation through an autoprocessing reaction (Fig. 11). In the case of Hh, its protein precursor undergoes autocatalytic cleavage, generating a 19 kDa N-terminal (N-Hh) and a 25 kDa C-terminal fragment (C-Hh). The C-terminus of N-Hh undergoes cholesterylation through a two-step autoprocessing reaction.^431^ First, the thiol side chain of Cys^257^ initiates a nucleophilic attack on the carbonyl carbon of Gly,^256^ triggering the cleavage of the Hh protein precursor and the formation of a labile thioester intermediate. Subsequently, cholesterol attacks the same carbon in the thioester intermediate, resulting in the cleavage of the intermediate as well as the formation of cholesterol-modified HhN and free HhC.^26^ During the process of cholesterylation, certain conserved amino acids of Hh act as vital factors. For instance, His,^328^ Thr^325^ and Cys^399^ are involved in thioester formation by forming hydrogen bonds with the α-amino group of Cys258 or by activating free thiols.^431,432^ Asp^302^ is needed for cholesterol attachment and 63 C-terminal amino acids are needed for the formation of a hydrophobic pocket to mediate cholesterol binding.^433^ In addition to the specific structure of HhC, the characteristics of cholesterol itself also contribute to the successful sterol attachment of Hh. The portion of the sterol that most likely acts as an attacking nucleophile is the 3β hydroxyl, and replacing the 3β hydroxyl with other groups, such as epicholesterol, would abolish autoprocessing, and the addition of hydroxy groups to the side chain or ring structures would result in decreased efficiency of the reaction.^433,434^ For SMO, the cholesterylation mechanism similarly involves a two-step process. The aspartic acid 95 (D95) and tyrosine 130 (Y130) of SMO join by an ester bond to form a high-energy intermediate, followed by ester exchange from D95–Y130 to D95–cholesterol.^435,436^ In addition to D95 and Y130, eight other residues, including E160, W109, W119, W163 and F166, are also essential for SMO cholesterylation. Replacing alanine with E160, F166, W109, W119, and W163 has been shown to entirely eliminate SMO cholesterylation.^437^ Moreover, the cation-π pair between Y85 and K133 is necessary for cholesterol conjugation to SMO.^437^ In addition to the specific structure of SMO, the N-Hh–PTCH1 axis increases [Ca2 + ] in SMO-localised endosomes, which further enhances SMO cholesterylation by promoting ester exchange from D95–Y130 to D95–cholesterol.^436^

### Role of cholesterylation

**Fig. 12:**
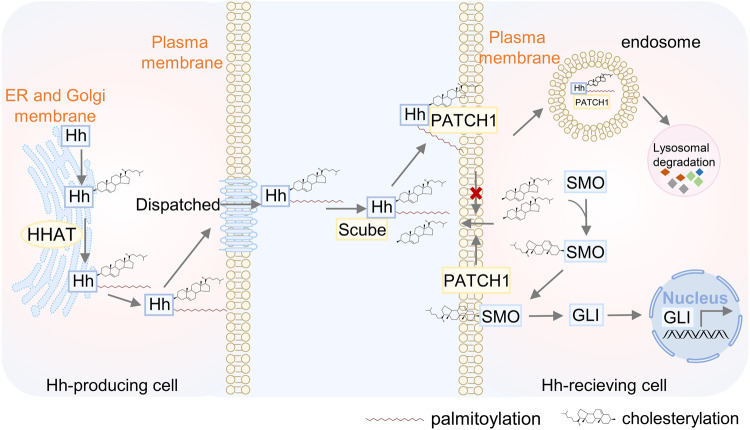
The role of cholesterylation in Hedgehog (Hh) signalling. The Hh protein undergoes cholesterylation and O-palmitoylation on the ER and Golgi membrane before targeting the plasma membrane (PM). Subsequently, it is secreted into the extracellular space and trafficked with the help of Dispathed and Scube. After arriving at the Hh-receiving cell, Hh binds to PATCH1, resulting in the degradation of PATCH1, which reduces cholesterol efflux and activates smoothened (SMO). Cholesterylated SMO traffics to the PM and then activates the downstream oncogenic GLI signalling pathway. ER endoplasmic reticulum

Hh signalling is a signal transduction pathway that regulates vital physiological activities such as embryonic development.^27^ Both N-Hh and SMO are critical components of the Hh signalling pathway, and cholesterylation has a pronounced effect on their functions (Fig. 12). In Hh-producing cells, N-Hh targets the PM after being covalently modified by cholesterol at the C-terminus and palmitate at the N-terminus, which is irreversibly catalysed by HAAT.^438^ Then, Dispatched, a multipass transmembrane protein located on the cell surface, controls the release of Hh into the extracellular space in a cholesterol-dependent binding event.^439^ The release of Hh is also dependent on Scube2, which is an extracellular protein assisting in the extracellular space trafficking of N-Hh.^439^ Although Dispatched and Scube2 cooperate to dramatically facilitate N-Hh release, the functional groups of cholesterol for recognition are different, and Dispatched is crosslinked 25-azicholesterol, while Scube2 is crosslinked with 6-azicholestanol.^439^ In Hh-receiving cells, the transmembrane protein PTCH1 is enriched in primary cilia and inhibits SMO activity before binding of Hh.^440^ Hh binding to PTCH1 facilitates endocytotic internalisation and the activation of N-Hh-PTCH1 complex degradation in lysosomes.^441^ Consequently, the inhibitory effect of PTCH1 on SMO is disarmed, and SMO becomes activated and relocates to the cilia, leading to activation of GLI transcription factors.^442^ Regarding SMO activation, exposure to cholesterol caused an enrichment of SMO at the cell surface, indicating that cholesterol is a candidate endogenous activator of SMO and needed for Hh signalling,^443^ as abolishment of cholesterylation by mutation of the D95 residue on SMO results in reduced Hh-stimulated ciliary localisation and compromised Hh signal transmission. Of the PM cholesterol pool consisting of accessible cholesterol (free cholesterol), sphingomyelin (SM) chelated cholesterol, and essential cholesterol, only free cholesterol is available for activation of Hh signalling.^444^ To further explore the relationship between PTCH1 and cholesterol concentration, it was found that fibroblast treatment with the Hh signalling antagonist cyclobenzaprine downregulated PTCH1 expression and reduced BODIPY cholesterol efflux, while the opposite phenomenon was observed when treated with the Hh signalling agonist SAG,^445^ suggesting that PTCH1 regulates intracellular cholesterol concentration by controlling cholesterol efflux from cells. In the absence of N-Hh, PTCH1 lowers the intracellular cholesterol concentration to inhibit the activation of SMO. In contrast, the interaction between N-Hh and PTCH1 creates a PTCH1-free ciliary environment for the further accumulation of free cholesterol, thereby contributing to SMO cholesterylation.

### Pathological implication

The Hh signalling pathway is pivotal in embryonic development.^441^ Regarding the impact of cholesterylation loss on development, abolishing cholesterylation of SMO was found to lead to embryonic death and severe developmental delay in homozygous SMO^D99E/D99E^ knockin mice.^436^ In addition, ShhN/- mutant mice unable to bind cholesterol exhibit developmental defects with phenotypic features similar to holoprosencephaly.^446^ Moreover, cholesterylation of N-Hh is essential to the development of limb buds; the role of the cholesterol moiety is to limit the spread of Shh across the anteroposterior axis, and it was observed that ShhN^−^ limbs lacking cholesterol modification have defective digit development.^447^ Taken together, the cholesterylation of N-Hh and SMO plays a pivotal role in regulating Hh signalling, and loss of cholesterylation disrupts Hh signalling and thus severely affects developmental patterning in various systems.

### Therapeutic targets and clinical research progress

Increased Hh pathway activity has pathological consequences in several cancer types,^448^ and targeting cholesterylation for the development of therapies against HH-related cancers maintains a promising outlook (Table 1). One of the strategies is targeting the autoprocessing of the Hh protein to block Hh cholesterylation and downstream signalling. Two compounds have been identified that effectively inhibit the cholesterol-dependent autocleavage of Hh proteins by targeting nucleophilic attack by cholesterol or cholesterol binding.^449^ CID 5717, also known as zafirlukast, inhibits cholesterol-dependent autocleavage in a time-dependent manner, which means that the inhibitory effect is reversible. CID 72303 was inhibited in a time-independent manner. The activity and safety of these compounds need to be further validated in vivo. In addition, reducing the biosynthesis of cholesterol is another strategy. Inhibition of HMG-CoA reductase by statins reduces cholesterol biosynthesis, markedly decreases Hh pathway activity and represses the proliferation of medulloblastoma cells.^314^ In addition, it is speculated that inhibition of sterol biosynthetic enzymes to divert sterol flux away from cholesterol into a “shunt” pathway may impair SMO activation by depleting cellular cholesterol, while more preclinical and clinical data are needed to support its application for SMO inhibition. Furthermore, SMO cholesterylation is regulated by SMO-interacting protein EBP. Overexpression of EBP suppresses SMO cholesterolization and downstream signalling, and the opposite phenomenon was observed after genetic disruption of EBP.^450^ Therefore, it may be considered therapeutically useful to upregulate EBP expression in diseases with enhanced Hh pathway activity and inhibit EBP expression in diseases with reduced Hh pathway activity.

## Conclusion and perspective

Diverse PTMs confer functional diversity to proteins. Since the discovery of the first protein lipidation in the 1970s, five decades of continuous investigations have led to a greater understanding of protein lipidation. Accumulating evidence from genetic, structural, and biomedical studies undoubtedly shows that lipidations play an important role in a wide range of physiological and pathological processes. Genetic evidence for lipidation-related enzymes in diseases and the role of lipidation in modulating protein functions are summarised in Tables 3 and 4.

**Table 3:** Genetic evidence for lipidation-related enzymes in diseases

Enzyme	Genetic evidence	Outcome	Ref.
DHHC1	Zdhhc1(−/−) mice	Zdhhc1(−/−) mice have lower cytokine levels and higher virus titre in the brain, indicating that DHHC1 is vital in mediating MITA/STING-dependent immune signalling against DNA viruses.	^475^
DHHC2	Zdhhc2(−/−) mice	Zdhhc2(−/−) mice inhibit plasmacytoid dendritic cell accumulation, impair T-cell activation and inhibit IFN-α production in the lesioned skin during psoriasis modelling, indicating that DHHC2 is vital in triggering immune response against inflammatory disorders.	^140^
DHHC3	Zdhhc3(−/−) mice	Binding of DHHC3 to UL20 is significant for virus infectivity and viral pathogenesis. HSV-1-infected Zdhhc3(−/−) mice have lower virus replication and decreased HSV-1 latency reactivation, indicating that the absence of DHHC3 blocks UL20 palmitoylation and then disturbs cytoplasmic envelopment of virions and virus egress.	^476^
DHHC3	Cardiomyocyte-specific transgenic mice overexpressing zDHHC3	DHHC3 promotes Rac1 signalling and maladaptive cardiac remodelling. Cardiomyocyte-specific transgenic mice overexpressing zDHHC3 exhibit enhanced Rac1 S-palmitoylation and downstream hypertrophic signalling, ultimately leading to cardiac disease.	^477^
DHHC4	Zdhhc4(−/−) mice	Palmitoylation of CD36 is meditated by DHHC4. Zdhhc4(−/−) mice exhibit decreased fatty acid uptake activity in adipose tissues and are intolerant to acute cold exposure, indicating that DHHC4 plays a crucial role in regulating fatty acid uptake by targeting CD36.	^87^
DHHC5	Zdhhc5^*flox/flox*^ mice	Palmitoylation of CD36 is also meditated by DHHC5. Zdhhc5^*flox/flox*^ mice also exhibit decreased fatty acid uptake activity in adipose tissues and are intolerant to acute cold exposure, indicating that DHHC5 also plays a crucial role in regulating fatty acid uptake by targeting CD36.	^87^
DHHC5	ZDHHC5 knockout mice (LysM-Cre /ZDHHC5^fl/fl^ mice)	Palmitoylation of NOD1/2 is meditated by DHHC5. LysM-Cre /ZDHHC5^fl/fl^ mice exhibit impaired NOD1/2–dependent activation of NF-kB and p38 MAPK signalling, indicating that palmitoylation of NOD1/2 meditated by DHHC5 is important in triggering immune response against peptidoglycans.	^144^
DHHC7	Zdhhc7(−/−) mice	Various synaptic and extrasynaptic proteins are meditated by DHHC7. Zdhhc7(−/−) mice exhibit impaired synaptic plasticity, while acute stress improves it only in females, but not in male mice, indicating that the role of DHHC7 in stress responses is sex-specific.	^478^
DHHC8	Zdhhc8(−/−) mice	DHHC8 deficiency is related to specific cognitive deficits and schizophrenia. Zdhhc8(−/−) mice exhibit impaired axonal growth, indicating that DHHC8 is important to modulate neuronal polarity.	^479^
DHHC9	Zdhhc9(−/−) mice	DHHC9 is associated with neurodevelopmental disorders. Zdhhc9(−/−) mice exhibit increased seizure activity and synaptic excitability, indicating that DHHC9 contributes to the pathogenesis of intellectual disability and epilepsy.	^480^
DHHC11	Zdhhc11(−/−) mice	DHHC11 is a positive regulator of DNA virus-triggered signalling. Zdhhc11(−/−) mice exhibit lower serum cytokine levels and are more sensitive to HSV-1-induced death, indicating that DHHC11 contributes to host defence against HSV-1 infection.	^481^
DHHC13	Transgenic DHHC13 mice	Palmitoylation of MC1R is meditated by DHHC13, which enhances DNA repair after ultraviolet irradiation. Transgenic DHHC13 mice have increased MC1R palmitoylation, rescue MC1R RHC-induced “red hair” phenotype and inhibit UVB-induced melanomagenesis in redheads, indicating that DHHC13-activated MC1R palmitoylation contributes to melanoma prevention.	^129^
DHHC13	Zdhhc13^skc4^ mice with a deficiency in DHHC13	Zdhhc13^skc4^ mice exhibit hyperproliferation of the epidermis and disturb cornification, cyclic alopecia and skin abnormalities, indicating that DHHC13 plays a crucial role in hair anchoring and skin barrier function.	^482^
DHHC15	Zdhhc15(−/−) mice	Genetic deficiency of DHHC15 is associated with intellectual disability and behavioural anomalies. Zdhhc15(−/−) mice exhibit increased tissue and extracellular dopamine levels in ventral striatum and novelty-induced locomotion in open field, indicating that DHHC15-mediated palmitoylation contributes to the regulation of dopamine in the striatum.	^483^
DHHC17	Zdhhc17(−/−) mice	DHHC17 regulates huntingtin protein and various synaptic protein. Zdhhc17(−/−) mice exhibit huntington disease-like neuropathology with corresponding behavioural, biochemical and neuropathological defects. Besides, behavioural and electrophysiological measures also suggest that Zdhhc17(−/−) mice have striatal dysfunction, astrogliosis and microgliosis, indicating that DHHC17 is essential for the maintenance of life and neuronal integrity.	^484,485^
DHHC19	Zdhhc19(−/−) mice	Zdhhc19−/− mice exhibit decreased testicular weight ratio, lower number and motility of the sperm and abnormal morphology. Zdhhc19 knockout male mice are sterile, and those results indicate that DHHC19 is vital in spermatogenesis and sperm functions.	^486,487^
DHHC21	Zdhhc21(−/−) mice	DHHC21 is involved in mediating signalling events required for gut hyperpermeability induced by inflammation. Zdhhc21(−/−) mice exhibit attenuated hyperpermeability response in an experimental model of thermal injury, indicating that targeting DHHC21 for burn-induced intestinal barrier dysfunction has therapeutic potential.	^488^
APT1	APT1 knockout mice	APT1 is able to depalmitoylate palmitoylated proteins implicated in exocytosis. APT1 knockout mice exhibit increased glucose-stimulated insulin secretion and β cell failure, indicating that APT1 is regulated in human islets and APT1 deficiency leads to β cell failure and type 2 diabetes.	^489^
NMT1	Heterozygous (+/−) Nmt1-deficient mice; Homozygous (−/−) Nmt1-deficient mouse	NMT1 plays an essential role in the early development of mouse embryo. Heterozygous (+/−) Nmt1-deficient mice exhibit suppressed macrophage colony forming and homozygous (−/−) Nmt1-deficient mouse embryonic stem cells exhibit a drastic reduction of macrophages after being stimulated by M-CSF, indicating that NMT1 is critical for proper monocytic differentiation.	^156^
FTase	FTase-deficient mice (FTΔ;RERTert/ert mice)	FTΔ;RERTert/ert mice exhibit delayed wound healing and maturation defects in erythroid cells. In tumour model, FTΔ;RERTert/ert mice exhibit reduced tumour development, which sheds light on the role of FTase in embryonic and tumour development.	^490^
FTase	Conditional FTase knockout mice (Fntb^fl/Δ^KL mice)	FTase-meditated farnesylation of HDJ2 and H-RAS promotes tumourigenesis. Fntb^fl/Δ^KL mice exhibit reduced tumour development and increased survival with K-RAS-induced lung cancer.	^345^
FTase	Keratinocyte-specific Fntb knockout mice (Fntb^Δ/Δ^ mice)	Fntb^Δ/Δ^ mice exhibit small and dysmorphic hair follicles and develop severe alopecia, while their skin barrier function is normal. Keratinocytes from Fntb^Δ/Δ^ mice are unable to proliferate, indicating that FTase is essential for the homeostasis of skin keratinocytes.	^491^
GGTase-I	Conditional FTase and GGTase-I knockout mice (Fntb^fl/Δ^Pggt1^bfl/Δ^KL mice)	Fntb^fl/Δ^Pggt1^bfl/Δ^ KL mice exhibit a far greater inhibitory effect on K-RAS-induced tumours than conditional FTase knockout mice, indicating that simultaneous inhibition of FTase and GGTase-I has a stronger antitumour effect and is therapeutically useful.	^345^
GGTase-I	Keratinocyte-specific Pggt1b knockout mice (Pggt1b^Δ/Δ^ mice)	Pggt1b^Δ/Δ^ mice exhibit stunted hair follicles and invariably die soon after birth. And keratinocytes from Pggt1b^Δ/Δ^ mice are unable to proliferate, indicating that GGTase-I is also essential for the homeostasis of skin keratinocytes.	^491^
GGTase-I	Podocyte-specific GGTase-I knockout mice	GGTase-I-mediated actin cytoskeleton is essential to maintaining podocyte function. Podocyte-specific GGTase-I knockout mice exhibit progressive albuminuria and foot process effacement due to dysregulation of the actin cytoskeleton, indicating that GGTase-I-mediated geranylgeranylation is crucial in the maintenance of glomerular integrity and function by regulating actin cytoskeleton.	^492^
GGTase-I	GGTase-I-deficient mice (Pggt1b^Δ/Δ^ mice)	Pggt1b^Δ/Δ^ mice exhibit enhanced inflammatory responses and severe rheumatoid arthritis, while Rac1 knockout prevents arthritis in GGTase-I-deficient mice, indicating that prenylation suppressed innate immune responses by blocking Rac1 effector interactions.	^275^
GGTase-I	GGTase-I-deficient mice (Pggt1b^fl/fl^Lyz2-Cre mice)	Geranylgeranylation involves in the antiviral innate immune response. Pggt1b^fl/fl^Lyz2-Cre mice exhibit improved survival upon lethal influenza A virus infection, indicating that impairment of protein geranylgeranylation contributes to part of the antiviral effect.	^267^
GGTase-I	T-cell-specific GGTase-I knockout mice (Pggt1b^ΔCD4^ mice)	GGTase-I-mediated prenylation regulates T-cell intestine localisation and chronic inflammation. Pggt1b^ΔCD4^ mice develop spontaneous colitis through impairing RHOA function and therefore increasing integrin alpha4beta7 expression as well as colon localisation, indicating that GGTase-I-mediated prenylation of RHOA is essential for its activation and colonic T-cell localisation.	^493^
GGTase-I	Pggt1b^−/−^ mice	Prenylation plays a crucial role in crthymocyte egress and immune homeostasis. Pggt1b^−/−^ mice exhibit marked defects in thymocyte egress and decreased T-cell lymphopenia in peripheral lymphoid organs, while Fntb − /− mice exhibit reduced percentages and numbers of PLN CD4+ and CD8 + T cells, revealing unique roles of prenylation in immune homeostasis mediated by GGTase-I or FTase, respectively.	^287^
PGAP4	PGAP4 knockout mice	PGAP4 catalyses the first step of GalNAc side chain generation. PGAP4 knockout mice exhibit abnormal bone formation, reduced locomotion activity as well as impaired memory formation, and they are more vulnerable to prion diseases, indicating that PGAP4-mediated GalNAc side chain is indispensable for various physiological function, especially in bone and the brain.	^494^
PGAP6	Pgap6 knockout mice	Pgap6 is involved in GPI-AP processing and regulates CRIPTO shedding. Pgap6 knockout mice exhibit defects in early embryonic development, especially anterior–posterior axis formation in embryos, indicating Pgap6-mediated CRIPTO shedding plays an essential role in embryonic development.	^495^

**Table 4:** The summary of the role of lipidation in modulating protein functions

Lipidation	Protein	The role of lipidation in modulating protein functions	Ref.
S-palmitoylation	CD36	S-palmitoylation increases CD36 intracellular trafficking and the incorporation of CD36 into the PM by enhancing its hydrophobicity.	^86^
	SCRIB	S-palmitoylation promotes SCRIB proper localisation to cell–cell junctions, and disrupting SCRIB S-palmitoylation by palmitoylation site mutation would cause a diffuse distribution of SCRIB in the cytosol.	^90^
	AMPAR	S-palmitoylation at TMD2 of AMPAR increases AMPAR internalisation and accumulation in Golgi, and depalmitoylation at this site promotes AMPAR to traffic from Golgi to the cell surface. The S-palmitoylation of C-terminal site regulates the interaction of AMPAR with 4.1N, and depalmitoylation increases the affinity with AMPAR-associated proteins such as 4.1N to keep AMPAR on the cell surface.	^92^
	FLT3	S-palmitoylation keeps FLT3 internal tandem repeat localising to ER, while disrupting FLT3 internal tandem repeat S-palmitoylation by palmitoylation site mutation would increase its PM localisation, followed by activation of Akt, ERK and STAT5.	^93^
	PD-L1	S-palmitoylation prevents PD-L1 from degradation. Blocking S-palmitoylation promotes its lysosomal degradation by inducing PD-L1 ubiquitination through the ESCRT-MVB pathway.	^98^
	Oct4	Oct4A is a variant of Oct4. S-palmitoylation prevents Oct4A from lysosome degradation to maintain its protein stability, thereby maintaining the stemness of Oct4-mediated glioma stem cells.	^100^
	NOD2	S-palmitoylation promotes NOD2 membrane recruitment and activation. Besides, S-palmitoylation prevents NOD2 from lysosome SQSTM1-mediated selective macroautophagic/autophagic degradation to maintain its protein stability.	^101^
	CLDN3	S-palmitoylation is required for CLDN3 proper PM localisation and its protein stability, thereby promoting ovarian cancer progress.	^102^
	spike	DHHC20 and DHHC9 are responsible for palmitoylation of spike proteins in ER and Golgi, following in company with viral budding to mediate viral fusion and infection of host cells.	^34,145^
	Fas	S-palmitoylation prevents Fas from lysosome degradation to maintain its protein stability, thereby promoting Fas-mediated apoptosis pathway.	^103^
	NLRP3	S-palmitoylation of NLRP3 promotes its chaperone-mediated autophagy and degradation, thereby impairing its protein stability and preventing sustained inflammation.	^104^
S-palmitoylation	AEG-1	S-palmitoylation of AEG-1 promotes its FBXW7-mediated ubiquitination and degradation. While disrupting AEG-1 S-palmitoylation by palmitoylation site mutation can weaken its binding to FBXW7 and increase its protein stability.	^83^
	IFNGR1	S-palmitoylation of IFNGR1 promotes its binding to AP3D1 and then lysosome degradation.	^105^
	CASP6	S-palmitoylation increases the flexibility of its loop 4 and enhances the interaction between loop 4 and loop 2, which leads to the blockage of activation site.	^108^
	PSD95	S-palmitoylation induces a conformational change of PSD95 from extended to compact, which leads to PSD95 binding directly to NMDAR and AMPAR subunits.	^109^
	STING	S-palmitoylation is required for STING activation in Golgi. Besides, STING S-palmitoylation enhances its interaction with VDAC2, thereby regulating tumour growth independent of innate immunity.	^110,112^
	cGAS	S-palmitoylation of cGAS reduces the interaction between cGAS and double-stranded DNA, thereby negatively regulating cGAS-mediated innate immune responses.	^116^
	STAT3	S-palmitoylation of STAT3 allows STAT3 to traffic and translocate to the PM for subsequent phosphorylation, and then phosphorylated STAT3 is depalmitoylated by APT2, which leads to STAT3 detaching from the PM and transporting to nucleus to activate the downstream genes RORC and IL17A, thereby initiating differentiation of TH17 cells.	^117^
	AGK	S-palmitoylation of AGK promotes translocation of AGK into the PM and activation of the PI3K-Akt-mTOR signalling pathway, thereby modulating sunitinib sensitivity against RCC.	^119^
	ACE2	S-palmitoylation and depalmitoylation of ACE2 is regulated by DHHC3 and APT1 for its PM targeting and extracellular vesicle secretion.	^147^
	GRK6	S-palmitoylation of GRK6 promotes translocation of GRK6 into the PM and activation of the PI3K-Akt signalling pathway, thereby promoting LPS-induced inflammatory response.	^120^
	PCSK9	S-palmitoylation of PCSK9 increases its affinity with PTEN and induces it to target lysosomes for degradation, thereby removing the inhibitory effect of PTEN on Akt signalling and leading to abnormal activation of Akt signalling.	^121^
S-palmitoylation	β-catenin	S-palmitoylation protects β-catenin againstβ-Trcp mediated proteasomal degradation and activates DUSP14 by upregulating c-Myc, thereby boosting CRC progression.	^124^
	MDH2	S-palmitoylation increases the activity of the key tricarboxylic acid cycle enzyme MDH2 to support mitochondrial respiration and tumour cell proliferation, thereby promoting the malignancy of ovarian cancer.	^125^
	GLUT1	S-palmitoylation promotes GLUT1 proper localisation to the PM for glucose transport, thereby assisting tumour cells in taking up nutrients to meet their high metabolic demands.	^126^
	HK1	S-palmitoylation of HK1 promotes its proper localisation to the PM in hepatic stellate cells. In the presence of TSG101, HK1 is secreted extracellularly via vesicles and further taken up by hepatocellular carcinoma cells to promote tumour cell glycolysis.	^127^
	MC1R	S-palmitoylation of MC1R RHC variant can rescue its function and activate MC1R signalling, thereby promoting pigmentation and controlling melanomagenesis.	^128^
	mTOR	S-palmitoylation of mTOR reduces its stability in a time-dependent manner, thereby disturbing the PI3K/Akt/mTOR signalling pathway, inhibiting breast cancer proliferation and improving its resistance to neratinib.	^131^
	syt11	S-palmitoylation of syt11promotes its localisation to the cell membrane rather than degradation in lysosomes, and this modification leads to enhanced binding of α-synuclein to the intracellular membranes and the abnormal aggregation of α-synuclein in PD neurons.	^135^
N-myristoylation	PKA	N-myristoylation of PKA promotes its membrane association by increasing its affinity with membranes.	^178^
	Gαi1	N-myristoylation of Gαi1 promotes its association with ordered lipid microdomains with higher phosphatidylserine content on the PM.	^185^
	FSP1	N-myristoylation of FSP1 promotes its membrane association and maintains its stability by evading the proteasome degradation pathway.	^186^
	VILIP3	N-myristoylation of VILIP3 protects itself from lysosomal pathway-mediated degradation, thereby enhancing its stability and subsequent NFκB/Bcl-2 signalling.	^187^
	Calcineurin	N-myristoylation of Calcineurin could increase its thermal stability.	^188^
N-myristoylation	c-Src	N-myristoylation of c-Src increases its association with ubiquitin E3 ligase and then promotes proteasome-mediated degradation of c-Src.	^192^
	p60v-src	N-myristoylation of p60v-src promotes its binding to receptor SLC25A5, thereby interacting with the membrane to keep the transformation activity of the virus.	^193,194^
	Lck	N-myristoylation of Lck promotes its proper localisation to the PM and then interaction with TCR complex, therefore initiating and propagating the TCR signalling cascade.	^496^
	HO-2	N-myristoylation of HO-2 is required for HO-2 binding to the myristic acid portion of Gag, and blocking HO-2 myristoylation would lead to an increase in viral replication.	^198^
	AMPK	N-myristoylation of AMPK is required for its lysosomal recruitment and activation, which suppresses overactivation of the mTORC1 pathway and T-cell differentiation into pro-inflammatory TH1 and TH17 helper T cells.	^209^
	ARF1	N-myristoylation of ARF1 is required for its function as a major regulator of STING membrane trafficking.	^205^
	Neurl-1	N-myristoylation of Neurl-1 is indispensable for the endocytosis and re-localisation on the PM of Notch ligand jagged 1.	^203^
	EZH2	Myristoylated EZH2 binds STAT3 to recruit it to phase-separated droplets, thereby overactivating STAT3 pathway to promote lung cancer progression.	^210^
	LAMTOR1	N-myristoylation of LAMTOR1 enhances its protein stability and lysosomal localisation, which leads to the activation of the mTORC1 pathway.	^211^
	ARF6	N-myristoylation of ARF6 facilitates its budding from the Golgi and its translocation in the GTP-bound form, thereby transporting EGFR from Golgi to the PM.	^213^
	Akt	Myristoylated Akt induces increased leptin levels in 3T3-L1 adipocytes, thereby involving in the development of obesity.	^217^
	MARCKS	N-myristoylation of MARCKS increases its affinity with cell membranes.	^177^
	Nef	N-myristoylation of Nef promotes its binding to the PM, which results in rapid internalisation of CD4 and MHC-1 of on the T-cell surface, leading to their degradation in lysosomes. Besides, through interaction with AP-1, myristoylated Nef disrupts the membrane delivery of VAMP3 and TNFα-positive endosome compartments and impairs optimal phagosome formation, thereby inhibiting macrophage phagocytosis.	^220–222^
	Gag	N-myristoylation of Gag on its N-terminal matrix domain promotes Gag targeting the PM and anchoring.	^195,196^
N-myristoylation	NKD2	N-myristoylation of NKD2 promotes its transport to the PM and interacting with Dvl-1, therefore leading to mutual ubiquitin-mediated proteasomal degradation and losing the ability to antagonise Wnt-beta-catenin activity.	^201,497^
	LYN	N-myristoylation of LYN promotes its proper localisation to the PM and subsequent phosphorylation, thereby interacting with BCR to regulate B-cell activation and function.	^498,499^
	HGAL	N-myristoylation of LYN promotes its proper localisation to cellular membrane raft microdomains, facilitating the interaction with SYK and modulation of the BCR activation and signalling.	^500^
S-prenylation	Ras	N-Ras, H-Ras, and K-Ras4a undergo prenylation and palmitoylation for anchoring to the PM via two hydrophobic moieties. Ras4b anchors to the PM through its farnesyl group combined with a lysine-rich polybasic sequence.	^266^
	MSPs	MSPs, such as MSP-1, MSP-2 and others are spread over the surface of Plasmodium merozoites, contributing to parasite survival and invasion.	^419^
	Rac1	Geranylgeranylation and subsequent palmitoylation of Rac1 is required for its translocation to the mitochondria-associated endoplasmic reticulum membrane (MAM), where Rac1 continues to limit the interaction of mitochondrial antiviral signalling proteins with Trim31, thereby inhibiting MAVS ubiquitination, aggregation and activation to prime antiviral immune response.	^267^
	CDC42	Geranylgeranyation of CDC42 is crucial for the interaction of CDC42 with its chaperone protein RHOGDI. The binding of RHOGDI to CDC42 not only facilitates correct membrane association of the Cdc42, but also protects it from degradation.	^277,278^
	Rheb	Farnesylation of Rheb overactivates mTORC1 signalling, and thus leading to cardiomyocyte hypertrophy.	^285^
	Rab	Dual geranylgeranylation of Rab is vital for the proper transport and localisation of Notch signalling elements. Incorrect trafficking of Notch signalling components caused by mislocalization of Rabs will in turns lead to the Notch signalling defects.	^288^
	Delta virus large antigen	Delta virus large antigen is prenylated, which is essential to anchor to the HBsAg and be packaged into virus particles for HDV particle formation.	^301^
	Ykt6	Farnesylation of Ykt6 induces a more compact and stable structure, thereby increasing its stability.	^276^
GPI anchor	FOLR1	The GPI-anchored protein FOLR1 mediates folate uptake for cell growth. When GPI-anchor of the folate receptors is replaced with transmembrane and cytosolic portions, the uptake efficiency of folate is significantly reduced. It also plays a significant role in modulating JAK-STAT3 signalling and ERK1/2 signalling.	^397–399^
	CD14	CD14 is a typical GPI-anchored glycoprotein. After recognising and binding to LPS, CD14 transfers LPS to MD-2 of the TLR4/MD-2 complex to promote TLR4 dimerisation. TLR4 dimerisation further induces the formation of Myddosomes based on MyD88, which triggers a signalling cascade, leading to activation of NF-κB and MAPK pathways.	^392,393^
	MSPs	MSPs, such as MSP-1, MSP-2 and others are spread over the surface of Plasmodium merozoites, contributing to parasite survival and invasion.	^419^
	PrPC	GPI is indispensable for PrPC to protect and repair neuronal cytoskeleton and neuronal communication.	^409^
	ART2	ART2 is a GPI-anchored exonuclease that uses free NAD+ to mono-ADP-ribosylate the P2X7 receptor on CD8 T cells, leading to NAD-induced cell death and a reduction of the P2X7R + CD8 + T-cell subpopulation in the tumour microenvironment.	^394,395^
	NCAM	NCAM is a typical adhesion molecule, which regulates cell–cell adhesion by homophilic and heterophilic interactions to regulate the development and plasticity of the nervous system.	^396^
	GPAA1	As a typical GPI-AP, GPAA1 enhances lipid raft formation to support the interaction between EGFR and ERBB2, which further activates downstream Akt signalling to enhance the proliferation of cancer cell.	^403^
	CD109	CD109 is a GPI glycoprotein that can be expressed on the cell surface and enriched in lipid rafts or released extracellularly with exosomes. CD109 involves in TGF‐β signalling, EGFR-Akt-mTOR signalling, JAK-STAT3 signalling and YAP/TAZ signalling.	^386,388,390^
	uPAR	As a typical GPI-AP, uPAP binds to uPA, and then uPA cleaves plasminogen to generate the active protease plasmin, which further activates MMPs to degrade ECM components and promote tumour cell migration and invasion. Besides, uPAR–α5β1 integrin interaction signals to FAK and then activates EGFR, and uPAR–β1 integrin–EGFR signalling enhances ERK activation to promote cancer cell proliferation.	^404,405^
GPI-anchor	CD55	CD55 is a complement regulator to prevent activated complement toxicity. The CD55-defective PNH red blood cells lack the self-protective complement regulatory factors, and thus are highly sensitive to complement activation, which results in intravascular haemolysis.	^407,408^
	CD59	CD59 is a complement regulator to prevent activated complement toxicity. The CD59-defective PNH red blood cells lack the self-protective complement regulatory factors, and thus are highly sensitive to complement activation, which results in intravascular haemolysis.	^407,408^
Cholesterylation	Hedgehog	Cholesterylation is required for Hedgehog to target the PM.	^438^
	SMO	Cholesterylation is required for SMO enriching at the cell surface and subsequent Hedehog signalling. Abolishment of cholesterylation by mutation of D95 residue on SMO results in reduced Hh-stimulated ciliary localisation and compromised Hedehog signal transduction.	^443^

*PM* plasma membrane, *ER* endoplasmic reticulum, *RCC* renal cell carcinoma, *CRC* colorectal cancer, *PD* Parkinson’s disease

We now have a comprehensive understanding of regulatory enzymes and catalytic processes involved in protein lipidation, as well as their role in physiology and disease. Based on this, several drugs have been developed and tested in preclinical and clinical studies, some of which including asciminib and lonafarnib are FDA-approved for therapeutic use. Nevertheless, given that lipidation involves delicate and complex regulation, there is still a fair amount of challenges for researchers to overcome. For instance, although chemical biology tools such as click chemistry-based metabolic labelling have facilitated the elucidation of protein lipidation, revealing the dynamic modification processes remains challenging. More convenient and intuitive detection techniques need to be further developed. In addition, some proteins (e.g., Ras) are not only dependent on S-palmitoylation and S-prenylation for their functions but also undergo other PTMs. How the crosstalk of protein PTMs is precisely orchestrated during this process remains unknown. There are more palmitoylation catalytic enzymes than other lipidation catalytic enzymes, and the understanding of the potential functional redundancy of DHHC-PATs in the S-palmitoylation machinery remains rudimentary, thus hindering the development of specifically targeted agents. Targeting DHHC remains an urgent need. Only a few crystal structures of DHHCs have been determined, and structure identification is required for guided rational drug design. To date, most DHHC inhibitors used in preclinical studies are broad-spectrum inhibitors. The design of selective inhibitors to pharmacologically control different DHHCs is an unsolved challenge. Whether DHHCs can serve as pharmacological targets for the treatment of human diseases needs more research. In addition, lipidation proteome analyses have revealed a large number of lipid-modified protein substrates, but most of them remain unannotated. Therefore, the functional impact on other proteins that undergo the same lipidation when using inhibitors or agonists of lipid modification is unknown. Substantial additional work is needed to elucidate the functional impact on these modified proteins. Moreover, revealing the three-dimensional structure of specific lipid-modified proteins of pathological importance to obtain valuable insights into substrate–enzyme relationships for further specialised inhibitor optimisation design may avoid off-target effects; however, this still requires tremendous effort. Drug resistance in the application of lipidation catalytic enzyme inhibitors is common. In addition to high-throughput screening and the development of novel small-molecule inhibitors, exploring potential drug synergies, especially targeting proteins that undergo multiple lipidations such as Ras, may improve drug resistance and therapeutic efficacy.

In summary, targeting lipidation in various diseases provides an innovative approach to achieve therapeutic goals, but it is fraught with challenges.
